# The intrinsically disordered protein SPE-56 is required for acrosomal-like exocytosis and fertility in *Caenorhabditis elegans*

**DOI:** 10.1038/s41598-026-47896-7

**Published:** 2026-04-11

**Authors:** Dieter-Christian Gottschling, Sarah Eiser, Frank Döring

**Affiliations:** Department of Molecular Prevention, University of Kiel, 24118 Kiel, Germany

**Keywords:** Fertility, Spermatogenesis, Acrosome reaction, Intrinsically disordered, *C. elegans*, Cell biology, Developmental biology, Genetics, Molecular biology, Physiology

## Abstract

In sexually reproducing organisms, activation of fertilization-competent sperm requires acrosomal vesicle exocytosis. We characterized the *C. elegans* gene *spe-56*, which encodes a single-pass transmembrane protein containing a C-terminal intrinsically disordered region (IDR). SPE-56 localizes to acrosomal-like membranous organelles (MOs) in the sperm, and loss of *spe-56* causes sterility due to defective MO–plasma membrane fusion during sperm activation. Genetic epistasis places SPE-56 downstream of the canonical SPE-8/SPE-6 sperm activation pathway, which comprises a membrane receptor and a kinase. Strains with partial deletions of the IDR are fertile at low temperatures but sterile at elevated temperatures. Larger deletions of the IDR are infertile at low and high temperatures. These data indicate that SPE-56 promotes acrosomal-like exocytosis and that its IDR contributes to the thermal robustness of sperm activation. Overall, IDR-mediated structural flexibility may represent a conserved mechanism for maintaining sperm function across variable temperatures.

**Supplementary Information:**

The online version contains supplementary material available at 10.1038/s41598-026-47896-7.

## Introduction

Sexual reproduction depends on the generation of haploid gametes through meiosis, a deeply conserved process that culminates in the production of spermatids in males across diverse taxa. During this process, known as spermatogenesis, spermatids undergo spermiogenesis, a complex maturation program that results in the maturation into motile, fertilization-competent spermatozoa^1–5^. Deciphering the molecular pathways that orchestrate sperm development and maturation is central to understanding the biological basis of fertility. Disruptions in key regulatory proteins - particularly transmembrane proteins - are frequently associated with sterile phenotypes, many of which are well documented in both humans and animal models^6–11^. However, despite this progress, the function of a subset of proteins that contain regions lacking stable tertiary structures remains poorly understood. These so-called intrinsically disordered proteins (IDPs) are characterized by conformational flexibility and dynamic structural plasticity, conferring unique functional capabilities. Dysregulation of IDPs has been implicated in a broad range of pathological processes^12,13^.

From a mechanistic perspective, IDPs are considered ‘edge of chaos’-systems that operate between order and complete randomness^14^. The functions of disordered regions in these proteins are often complementary to those of ordered protein domains, which are typically involved in regulation, signaling and control pathways^15,16^. ID domains comprising 30 or more consecutive residues constitute 13.3% and 3.5% of human plasma membrane proteins on the inside and outside of cells, respectively, indicating a cytosolic preference^17^.

Comprehensive functional proteomics analyses in mammals revealed that intrinsically disordered domains of transmembrane proteins act as highly tunable modulators of membrane curvature and remodeling^18^. Membrane remodeling and transient destabilization are critical for numerous cellular processes, including endocytosis, exocytosis, and specialized events such as the acrosome reaction in sperm or sperm–oocyte fusion. These processes require rapid reorganization of membrane proteins and phospholipids to facilitate membrane fusion, content release, and the formation of fusion pores^19,20^. Remarkably, these membrane-destabilizing factors can confer fusogenic properties while preserving overall membrane integrity, ensuring optimal function during these critical events^21,22^.

The conserved functions of IDPs have also been described in several animal models, including *Drosophila* fruit flies^23^, *Mus musculus* mice^24^, and *C. elegans* nematodes^25^. In *C. elegans*, IDPs have been implicated in several basic biological processes, such as the regulation of *pi*RNA-induced gene silencing, a critical mechanism for maintaining germline integrity and immortality^25^. Intrinsically disordered regions (IDRs) within these proteins have also been shown to regulate the dynamic behavior of germ plasm components, which is essential for normal embryogenesis^26^. In addition, the sperm-specific IDPs WAGO-3 and SPE-18 play critical roles in fertility by facilitating meiotic maturation during the early stages of sperm development^27,28^. However, a broader functional repertoire of IDRs involved in *C. elegans* spermatogenesis, specifically in regulation of spermiogenesis remains elusive.

Owing to its defined anatomy, genetic tractability, and conserved pathways, the nematode *Caenorhabditis elegans* has emerged as a widely used model organism for basic biological research^29–31^. Its reproductive system provides a robust framework for dissecting the molecular and cellular mechanisms underlying fertility. *C. elegans* has two sexes: hermaphrodites and males. Both sexes are characterized by the production of sperm (spermatogenesis) through two distinct sequential processes: the differentiation of spermatocytes, including their meiotic maturation into spermatids, and the activation of spermatids into motile, fertilization-competent spermatozoa (spermiogenesis)^11,32^.

In *C. elegans*, spermatogenesis occurs in both sexes and is initiated by the differentiation of spermatocytes from spermatogonial precursor cells. Primary spermatocytes enter meiosis and further differentiate into secondary spermatocytes that proceed through a second meiotic division to produce four haploid spermatids^33,34^. This last step of meiotic differentiation is accompanied by the formation of a residual body (RB), into which superfluous cytoplasmic components are sequestered and subsequently discarded^35,36^. Conversely, essential elements such as Golgi-derived membranous organelles (MOs) and proteins that are critical for spermiogenesis and fertilization are selectively retained within spermatids that subsequently undergo spermiogenesis^32^. Spermiogenesis in *C. elegans* is defined as the maturation of round, immotile spermatids into motile, bipolar spermatozoa with the abilities to move and fertilize. This process involves two hallmark-events: (1) The MO fusion process with the plasma membrane which is analogous to the acrosome reaction^37^ and (2) Acquisition of sperm motility through the formation, extension and treadmilling of a pseudopod which is analogous of the metabolic changes and motility changes of other sperm^32,38^. The MO fusion process generates stable fusion pores at the sperm head and facilitates the release of key fertilization factors, including SPE-9 and SPE-36, which are essential for sperm–oocyte interactions^11,39^, paternal-effect embryonic lethal factors like SPE-11 necessary for embryogenesis^40^, and the MO transmembrane toxin PEEL-1 that is delivered to the oocyte during fertilization and, subsequently to the embryo, where it is required for speciation^41^.

In *C. elegans* hermaphrodites, spermiogenesis is regulated by the SPE-8 signaling pathway, including the proteins SPE-8, SPE-12, SPE-19, SPE-27, SPE-29, and SPE-43, which mediate the activation of spermatids instantly in response to seminal stimuli^42–44^. Conversely, in males, instant activation is prevented by the TRY-5 inhibitor SWM-1 before mating, allowing spermatids to be stored in a quiescent state until transfer during mating with hermaphrodites^45^. While both the SPE-8 and TRY-5 pathways are functional in males, only the SPE-8 pathway has been demonstrated to be functional in hermaphrodites. However, both pathways preserve the same crucial steps, such as MO fusion and pseudopod extension, required for activation. In past years, a significant body of research has been conducted investigating these complex events that define spermatogenesis^32,37,43,45,46^. However, no IDR-containing proteins have been described yet as participating in spermiogenesis.

In this study, we identified SPE-56, a previously uncharacterized sperm-specific single-pass transmembrane IDP that localizes to the MOs of *C. elegans* sperm. Functional analyses revealed that SPE-56 is essential for the execution of spermiogenesis, specifically by mediating both MO-PM fusion and the extension of the initially short pseudopods - two critical steps required for spermiogenesis.

## Results

### *F56D5.2* encodes a novel protein necessary for hermaphrodite self-fertility

**Fig. 1:**
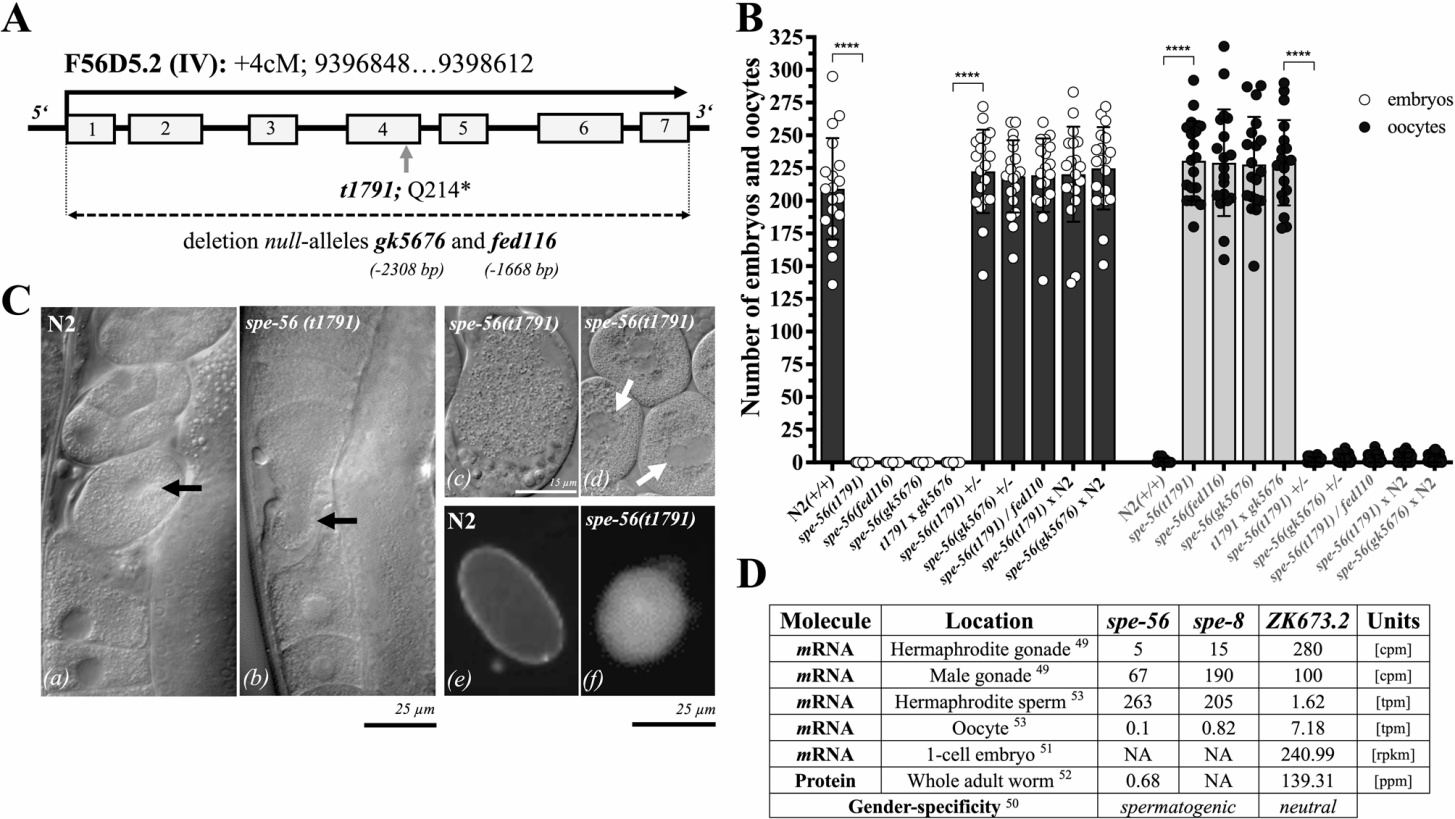
Structure and fertility phenotypes of *F56D5.2* mutant alleles. **(A)** Schematic of the *F56D5.2* gene structure showing the *t1791* allele (gray arrow), which results in a premature STOP (*) codon at Q214, and the CRISPR/Cas9-generated *null*-alleles *gk5676* and *fed116* encompassing the deletion of the entire *F56D5.2* genomic sequence. **(B)** Numbers of embryos and unfertilized oocytes produced by *F56D5.2* homozygous and heterozygous mutant hermaphrodites, including rescued and trans-heterozygous animals. The values represent the means (± SD) of *N* = 3 independent experiments involving *n* ≥ 10 animals per trial. *p < 0.0332; **p < 0.0021; ***p < 0.0002; ****p < 0.0001 (Ordinary one-way ANOVA). **(C)** Differential interference contrast (DIC) and fluorescence images depicting the uterus of a wild-type (N2) hermaphrodite harboring fertilized oocytes and early embryos ***(a)*** compared with the uterus of a *t1791* mutant hermaphrodite filled with unfertilized ***(b)*** and endomitotic oocytes (EMOs) ***(c***,*** d)***. Oocytes are indicated by black arrows. White arrows indicate multiple endomitotic nuclei. *Trypan-blue* staining reveals abnormal membrane permeability of EMOs in *t1791* mutants ***(f)*** compared to fertilized N2 wild-type oocytes enclosed by an eggshell ***(e)***. **(D)** Expression profiles of *spe-56*, *spe-8*, and *ZK673.2* based on transcriptomic and proteomic data. *spe-56 m*RNA is primarily found in sperm. The units are “counts per million” (cpm), “transcripts per million” (tpm), “reads per kilobase million” (rpkm), and “parts per million” (ppm).

Previous studies on whole-genome sequencing of maternal-effect lethal and sterile *C. elegans* mutants resulted in the identification of two alleles, *t1744* (S109F) and *t1791* (Q214stop), of the *F56D5.2* gene. This gene encodes an novel protein that is essential for sperm function^47,48^. Here, we functionally characterize F56D5.2 using null alleles (*gk5676*,* fed116*) and the *t1791* allele. Homozygous *t1791* and *gk5676* hermaphrodites exhibited a fully penetrant self-sterile phenotype compared to wild-type hermaphrodites. On average, they laid 200 unfertilized oocytes but no viable progeny. In contrast, wild-type hermaphrodites produced an average of 200 viable progeny (Fig. 1B). Notably, the *t1791/gk5676* trans-heterozygotes produced self-sterile progeny that were phenotypically indistinguishable from the corresponding homozygous single mutants, indicating that these alleles fail to complement and are likely to disrupt the same gene (Fig. 1A, B).

All characteristics described were confirmed by *fed116*, generated by CRISPR/Cas9 deletion (−1668 bp) of the entire *F56D5.2* genomic sequence (Fig. 1A, B). Strikingly, homozygous integration of a single copy of wild-type *F56D5.2* via *Mos1*-mediated insertion fully rescued the self-sterility of *t1791* animals, confirming the gene’s molecular identity and function (Fig. 1B). For further analysis, the alleles *t1791*, *gk5676*, and *fed116* were classified as recessive *null* alleles.

### *F56D5.2* is a member of the “*spe”* gene family required for sperm function

Detailed stereomicroscopic examination of *F56D5.2* mutant hermaphrodites and males revealed no discernible differences compared to wild-type animals. The developmental timing, lifespan, and locomotor activity (crawling and swimming) of *F56D5.2* mutants were indistinguishable from those of N2 wild types, suggesting that *F56D5.2* is not involved in essential somatic functions under standard laboratory conditions. Examination of the gonadal structures - including the germline, spermatheca, and mature oocytes - revealed no obvious morphological defects in the hermaphrodite mutants (data and images are available upon request).

However, despite normal oogenesis (Fig. 1C a, b), mutant hermaphrodites accumulated endomitotic oocytes (EMOs) in the uterus but not the oviduct. These EMOs are characterized by abnormal round morphology, multinucleation (Fig. 1C c, d), and trypan-blue permeability (Fig. 1C e, f), which is indicative of failed fertilization. Importantly, *F56D5.2* mutant hermaphrodites produced abundant viable progeny when mated with N2 wild-type males and did not accumulate unfertilized oocytes under these conditions (Fig. 1B). These results indicate that *F56D5.2* mutant hermaphrodites are self-sterile but retain full cross-fertility, indicating a defect specific to sperm. Consistent with this interpretation, transcriptome profiling revealed that *F56D5.2* mRNA is expressed in spermatocytes, with no detectable expression in oocytes or early embryos^49–53^. Proteomic analyses further identified *F56D5.2* as a low-abundance sperm-specific protein (Fig. 1D). Notably, *F56D5.2* is located within a characterized sperm gene cluster on chromosome IV at + 4.24 cM, reinforcing its assignment to the sperm gene regulatory network.

Together, these findings indicate that *F56D5.2* is required for sperm function, which is consistent with the phenotypic and molecular characteristics of previously described *spe* (SPErmatogenesis-defective) genes. Accordingly, we designate *F56D5.2* as *spe-56*, in agreement with the standard nomenclature.

### *spe-56*-deficient males are sterile but retain mating competence, sperm transfer ability, and transactivation potential

**Fig. 2:**
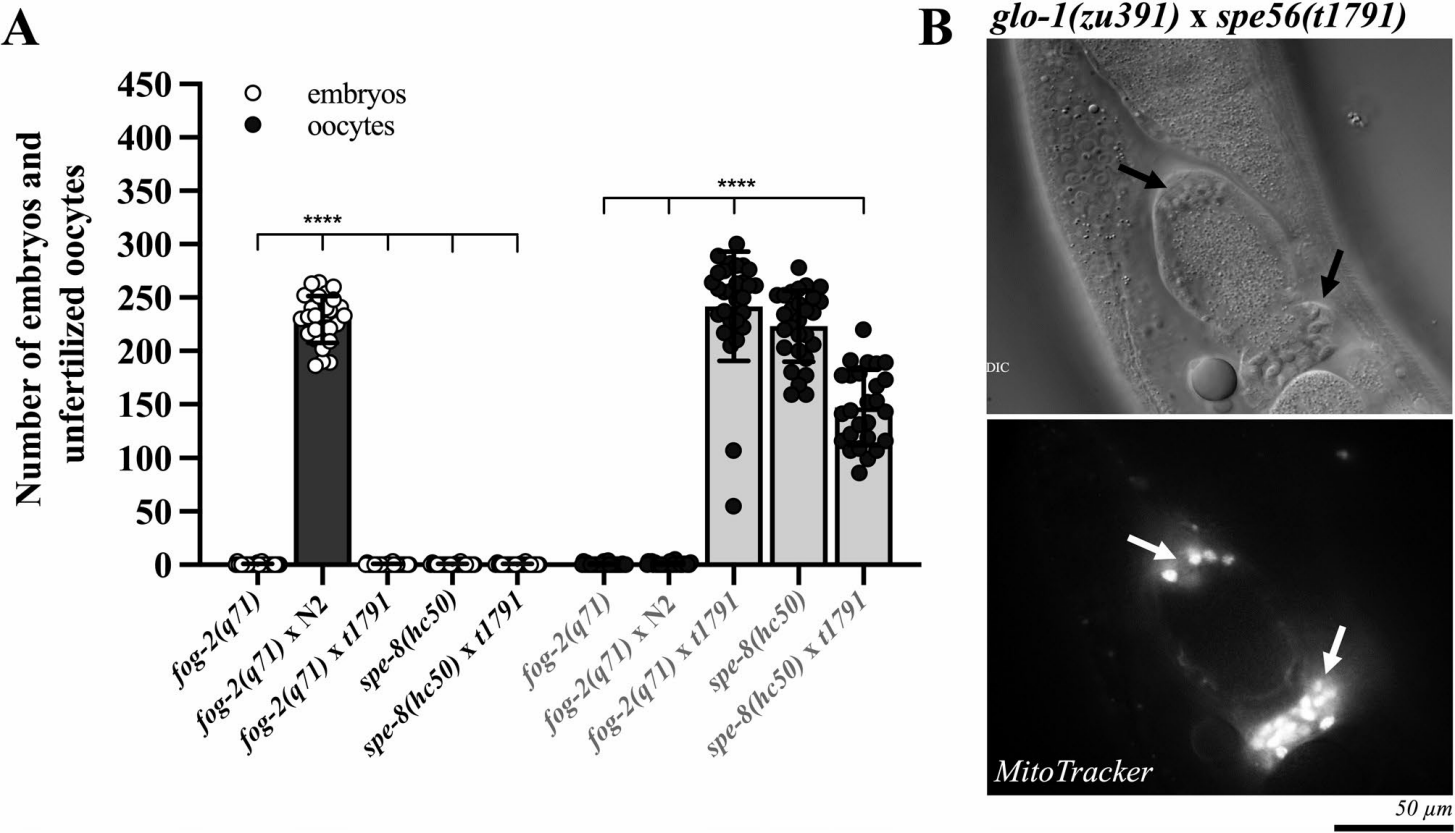
In vivo analysis of fertility, sperm transfer, and trans-activation. **(A)** Quantification of embryos and unfertilized oocytes produced by *fog-2(q71)* and *spe-8* mutant hermaphrodites following mated with either wild-type (N2) or *spe-56(t1791)* mutant males. The values represent the means (± SD) of *N* = 3 independent experiments involving *n* ≥ 10 animals per trial. *p < 0.0332; **p < 0.0021; ***p < 0.0002; ****p < 0.0001 (Ordinary one-way ANOVA). **(B)** DIC and fluorescence images of a hermaphrodite carrying the *glo-1(zu391)* allele, which reduces background autofluorescence, showing transferred sperm (indicated by arrows) from *spe-56*-deficient males in the spermatheca.

To assess male fertility, we crossed *spe-56(t1791)* males with *fog-2(q71)* hermaphrodites, which lack endogenous sperm and consequently do not produce either self-progeny or unfertilized oocytes (Fig. 2A). While wild-type males reliably restore fertility in *fog-2(q71)* spermless hermaphrodites, resulting in abundant cross-progeny, *spe-56*-deficient males fail to sire any offspring (Fig. 2A). However, despite their complete sterility, *spe-56(t1791)* males were fully capable of mating. This is evident from successful sperm transfer (Fig. 2B) and the induction of oocyte production in *fog-2(q71)* (Fig. 2A), indicating that biologically functional seminal fluid was transferred. Consistent with normal mating behavior, *MitoTracker*-labeled^54,55^ sperm from *spe-56*-deficient males were detected in close contact to oocytes after mating with *glo-1(zu391)* hermaphrodites, which lack auto-fluorescence in their guts (Fig. 2B)^56^. These observations suggest that sterility in *spe-56*-deficient males is not due to a defect in mating, sperm transfer, or transit to the site of fertilization but rather to a functional defect in sperm.

To determine whether this defect might be the result of impaired seminal fluid–mediated activation of sperm, we employed *spe-8(hc50)* mutant hermaphrodites, which are known to lack self-fertility unless their sperm are trans-activated by male-derived seminal fluid. Strikingly, *spe-56-*deficient males restored the fertility of *spe-8*-deficient hermaphrodites without generating outcross progeny, demonstrating that their seminal fluid retains the capacity to induce sperm activation in ‘trans’ (Fig. 2A).

In conclusion, these results suggest that the sterility of *spe-56*-deficient males arises from an intrinsic functional defect in sperm rather than from impaired mating behavior, sperm transfer, or seminal fluid function.

### *spe-56*-deficient spermatozoa form stubby pseudopods and fail to maintain position in the spermatheca

**Fig. 3:**
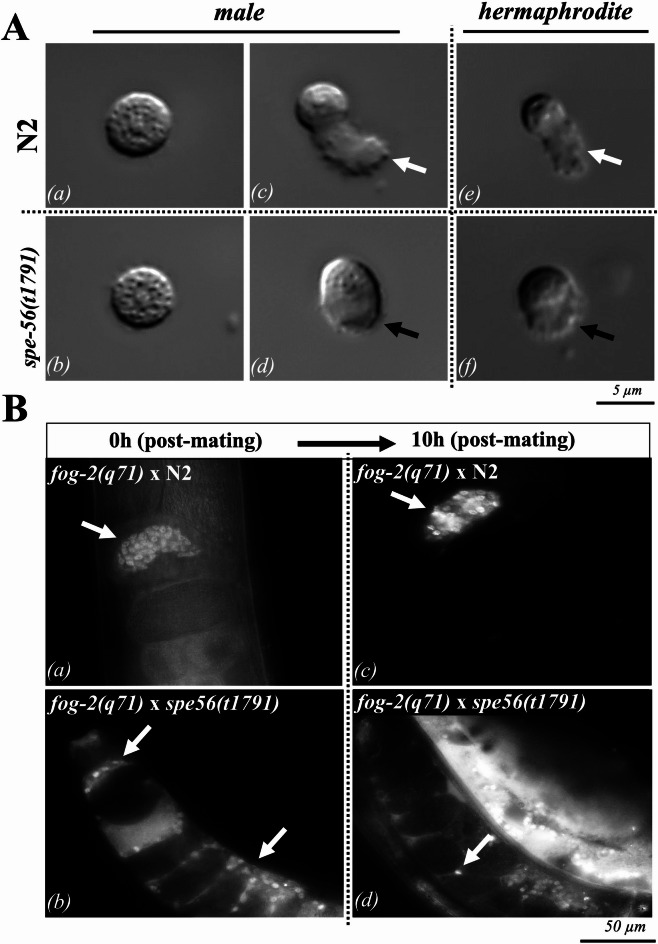
Morphology and in vivo dynamics of *spe-56*-deficient sperm. **(A)** Differential interference contrast (DIC) images of spermatids ***(a***,*** b)***, in vitro-activated spermatozoa from males ***(c***,*** d)***, and in vivo-activated spermatozoa from N2 wild-type ***(e)***, and *spe-56(t1791)* mutants ***(f)***. Wild-type sperm show a hemispherical cell body with dynamic pseudopods (white arrows), whereas *spe-56* mutant sperm exhibit abnormally short pseudopods (black arrows). (**B)** Fluorescence images illustrate the distribution of *MitoTracker* labeled sperm in *fog-2(q71)* hermaphrodites carrying spermatozoa from N2 ***(a)***, and *spe-56(t1791)* ***(b)*** males. The localization of sperm in *fog-2(q71)* is shown at two points: immediately (0 h) and 10 h post-mating. ***(c***,*** d)***. Arrows indicate sperm position, revealing stable localization of wild-type sperm within the spermatheca, in contrast to randomized displacement of *spe-56* mutant sperm throughout the uterus or beyond.

During spermiogenesis, sessile spermatids are activated by seminal fluid into motile spermatozoa capable of fertilization. DIC microscopy revealed that *spe-56(t1791)* spermatids were morphologically indistinguishable from wild-type spermatids and presented a characteristic round shape and central nucleus (Fig. 3A, a, b). Following in vivo activation, wild-type sperm developed dynamic, hemispherical pseudopods essential for rapid amoeboid movement (Fig. 3A, c, e). In contrast, activated *spe-56(t1791)* spermatozoa from both males and hermaphrodites presented shorter, less dynamic pseudopods despite the formation of a bipolar cell body (Fig. 3A, d, f).

To examine sperm motility in vivo, we mated *spe-56(t1791)* males carrying *MitoTracker*-labeled sperm, with *fog-2(q71)* hermaphrodites and tracked the labeled sperm over time. Remarkably, while wild-type sperm actively crawled back into the spermatheca after oocyte passage and displacement, *spe-56*-deficient sperm failed to find the way back to the spermatheca; instead, they randomly accumulated in the uterus or were expelled (Fig. 3B, b, d). These observations demonstrate that *spe-56* sperm are less motile and unable to maintain their position in the spermatheca over time. This is likely due to defective pseudopod morphology, resulting in reduced crawling efficiency. Despite this impairment, a small number of *spe-56*-deficient sperm have been found to persist in the spermatheca long enough to come into contact with oocytes. This implies that sterility may arise from an additional defect intrinsic to the sperm that alters the fertilization process itself. The on-going laying of unfertilized oocytes suggests that the mutant sperm is continuing to stimulate ovulation, indicating the presence of functional Major Sperm Protein (MSP) which is required for oocyte maturation^57^.

### SPE-56 is required for MO-PM fusion during sperm activation

During sperm activation, membranous organelles (MOs) fuse with the plasma membrane (PM), delivering key factors essential for fertilization. This specialized form of exocytosis is analogous to the acrosome reaction in mammalian sperm.

**Fig. 4:**
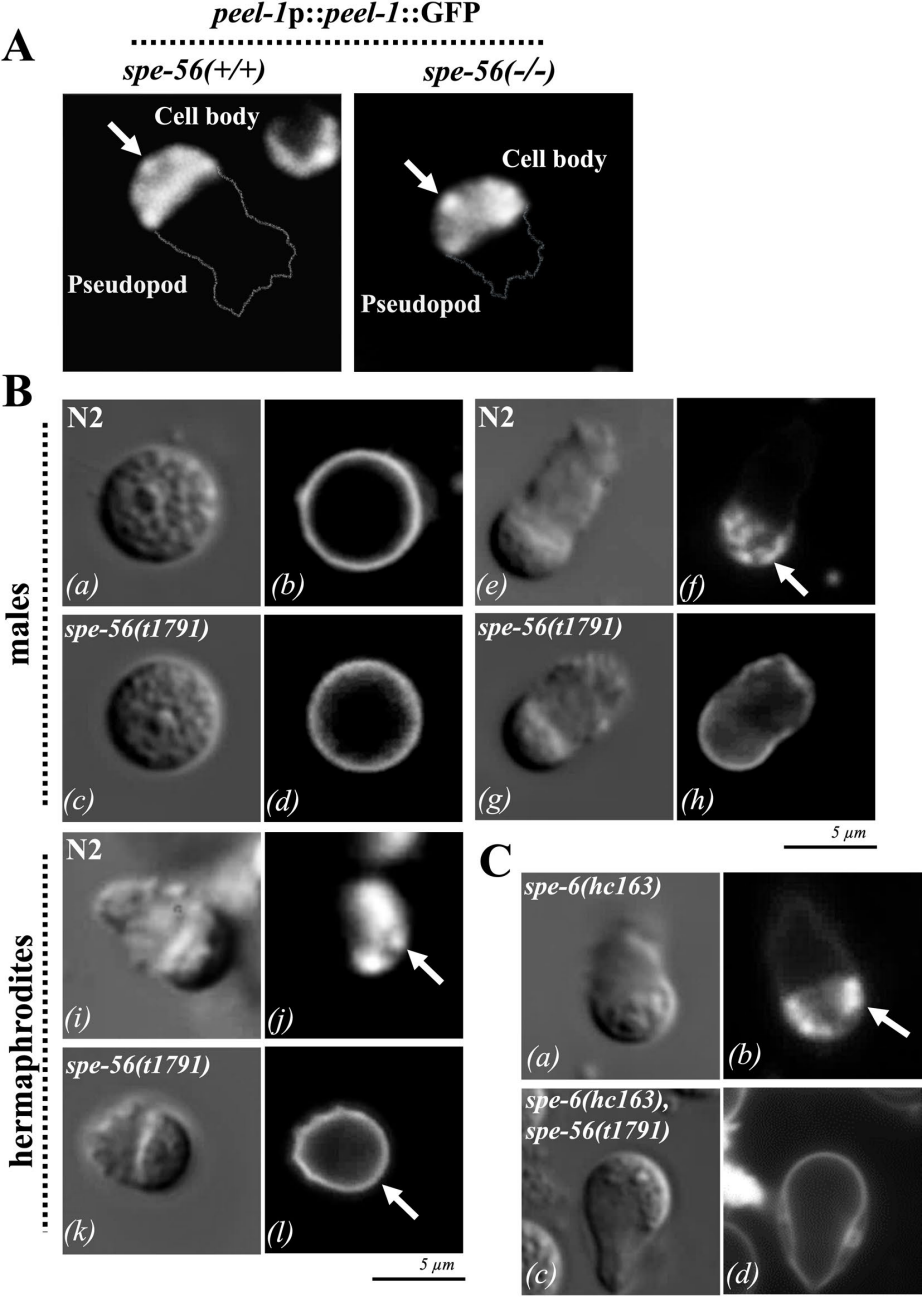
Evaluation of sperm activation in *spe-56* deficient mutants. **(A)** Fluorescence images of N2 wild-type (+/+) and *spe-56* (–/–) spermatozoa expressing a PEEL-1::GFP fusion protein^41^, which localizes to membranous organelles (MOs) at the sperm head. White arrows indicate bright puncta corresponding to MO-associated PEEL-1::GFP. **(B)** DIC and fluorescence images of spermatids ***(a–d)*** and spermatozoa ***(e–l)*** isolated from N2 wild-type and *spe-56(t1791)* males ***(a–h)*** and hermaphrodites ***(i–l)*** stained with the lipophilic dye FM1-43^46^^108^,. White arrows mark fusion pores (bright puncta) resulting from MO–plasma membrane (PM) fusion, indicating successful sperm activation. **(C)** DIC and fluorescence images of FM1-43–stained *spe-6(hc163)* constitutively activated mutant spermatozoa, and spermatozoa from *spe-6(hc163)*,*spe-56(t1791)* double mutants. White arrows highlight MO–PM fusion pores (bright puncta), which indicate the activation status.

In the *spe-56(t1791)* genetic background, the utilization of a PEEL-1::GFP reporter to trace the membrane protein fate of the MOs proved inconclusive with regard to the state of MO-PM fusion following sperm activation^41,58^. Noteworthy, the stubby *spe-56(t1791)* pseudopods were clearly detectable in the *spe-56(t1791)*,*peel-1(oxSi78)* double mutants, as compared to *peel-1(oxSi78)* controls with normal pseudopods (Fig. 4A).

To assess MO–PM fusion more directly, we employed the lipophilic membrane dye FM 1–43, which integrates into the outer leaflet of the PM. In spermatids from both N2 wild-type and *spe-56(t1791)* males, FM 1–43 was evenly distributed across the PM (Fig. 4B, a–d). When wild-type male (Fig. 4B, e, f) or hermaphrodite (Fig. 4B, i, j) sperm are activated, MO fusion produces stable PM invaginations that are observed as bright FM 1–43-labeled puncta. In contrast, activated *spe-56(t1791)* sperm from both males (Fig. 4B, g, h) and hermaphrodites (Fig. 4B, k, l) displayed a uniform FM 1–43 signal. They lacked the characteristic PM invaginations, indicating an inability to initiate or complete MO fusion.

Overall, these findings demonstrate that SPE-56 is essential for promoting MO–PM fusion during sperm activation, a critical step in preparing sperm for fertilization.

### SPE-56 functions downstream of the canonical SPE-8/SPE-6 sperm activation pathway in vivo

In *C. elegans* hermaphrodites, sperm activation is initiated by the SPE-8 signaling pathway, which comprises the membrane-associated proteins SPE-8, SPE-12, SPE-19, SPE-27, and SPE-29 encoded by the *spe-8*-group of genes. These proteins transduce an extracellular activation signal derived from seminal fluid factors. Loss-of-function mutations in *spe-8*-group genes are known to disrupt sperm activation in hermaphrodites^59–61^. Downstream of this pathway, the casein kinase SPE-6 acts as a negative regulator, preventing precocious activation of spermatids in the absence of an activation signal. Reduction-of-function alleles of *spe-6*, such as *hc163*, can bypass the transduction of activation signals through the SPE-8 cascade, partially restoring sperm activation^62–64^.

To determine the position of *spe-56* within this activation pathway, we used genetic epistasis with *spe-6(hc163)* and *spe-56(t1791)* double mutants. As expected, wild-type males produced quiescent spermatids, whereas *spe-6(hc163)* males generated prematurely activated spermatozoa, characterized by fully extended pseudopods and successful MO fusion (Fig. 4C, a, b). In contrast, the sperm of the *spe-6(hc163)*,*spe-56(t1791)* double mutants were indistinguishable from those of the *spe-56(t1791)* single mutants. Both displayed stubby pseudopods and were unable to undergo MO fusion (Fig. 4B, g, h, C, c and d).

These results indicate that *spe-56* acts downstream of *spe-6* in the sperm activation pathway and is required to mediate critical cellular events, such as MO fusion and pseudopod extension, which are essential for successful sperm activation.

### SPE-56 facilitates a terminal step in sperm activation in vitro

Spermatid activation into motile spermatozoa can be induced in vitro via various stimuli that mimic distinct stages of spermiogenesis. The weak base triethanolamine (TEA) and the cationic ionophore Monensin are known to increase the intracellular *p*H and promote activation (Fig. S1 a-h)^65^. In contrast, the protease Pronase specifically activates SPE-8 signaling without altering the internal *p*H (Fig. S1 m-p)^66^. Furthermore, Zinc, a micronutrient, functions downstream of the SPE-8 group (Fig. S1 m-u), whereas Proteinase-K triggers activation independently of SPE-8 (Fig. S1 i-l)^43^^[,67^.

To reveal the functional context of SPE-56, we tested sperm activation in vitro using the stimuli described above. N2 wild-type spermatids responded robustly to all five stimuli, exhibiting characteristic features of activation, including MO fusion and normal pseudopod extension. In contrast, *spe-56*-deficient spermatids displayed only partial activation in response to each treatment, forming abnormally short pseudopods and failing to undergo MO–PM fusion (Fig. S1 a-u).

These findings are in line with our in vivo data and demonstrate that SPE-56 acts downstream of the canonical SPE-8/SPE-6 pathway. Specifically, SPE-56 is required for executing at least one terminal event in sperm activation, including MO fusion and pseudopod extension. Thus, SPE-56 functions downstream of all the in vitro activators that have been described (Fig. S1), rendering sperm competent for fertilization.

### SPE-56 localizes to MOs in spermatids and spermatozoa

Upon activation, MOs fuse with the PM forming permanent fusion pores that maintain connectivity between the MO and the PM. Consequently, the membranes of the fused MOs become integrated into the PM. During this process, MO-associated transmembrane proteins are either incorporated into the PM (e.g., the sperm receptor SPE-9) or retained within the fused MO membrane (e.g., the Ferlin protein FER-1)^11^^[,46^.

**Fig. 5:**
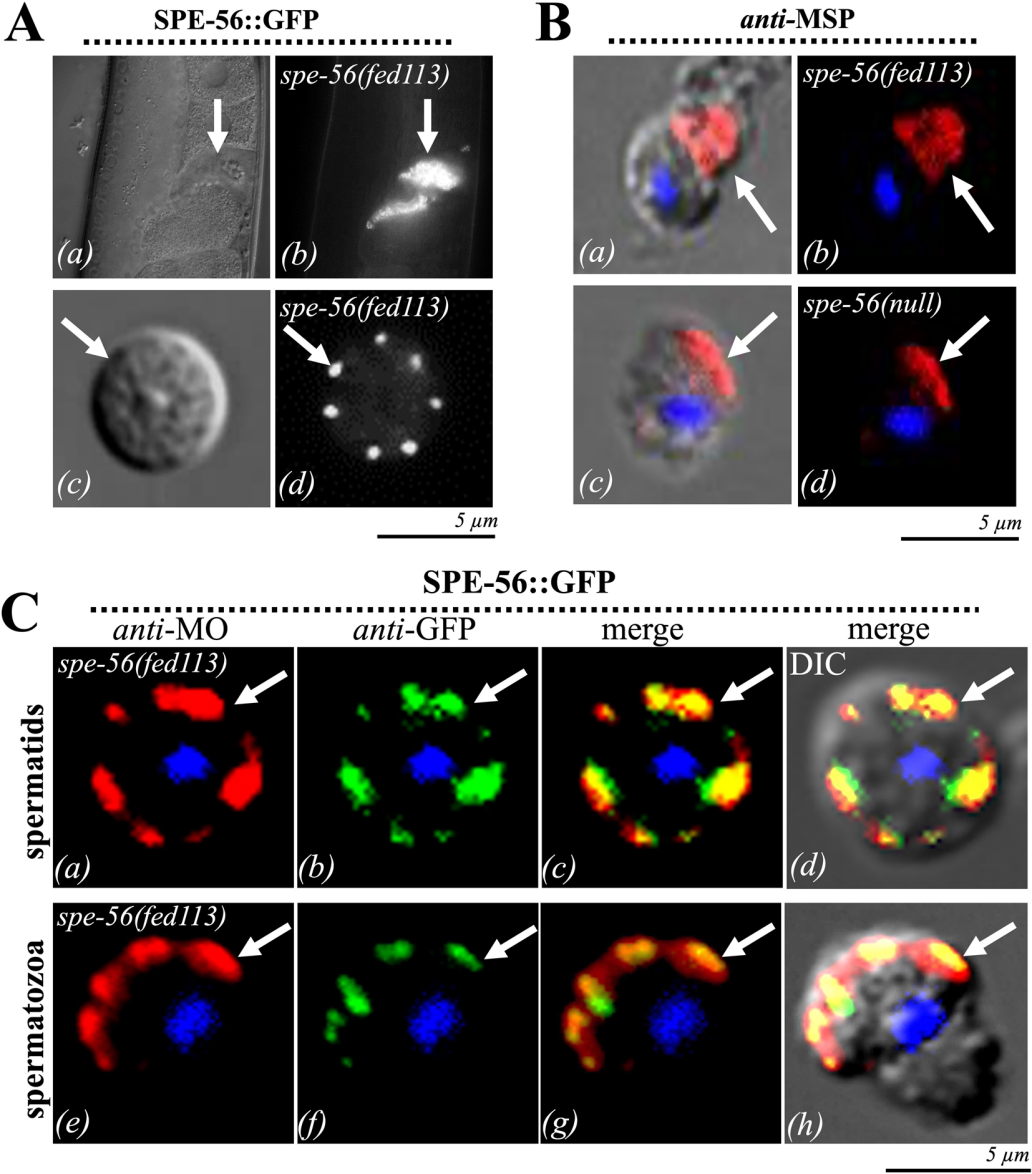
Localization of SPE-56 at the cellular and subcellular levels. **(A)** DIC and fluorescence in vivo images of **(a)** sperm within the spermatheca of *spe-56(fed113)* hermaphrodites expressing a C-terminally tagged SPE-56::GFP fusion protein from the endogenous *spe-56* locus. Arrows indicate GFP-positive sperm. ***(b)*** In vitro DIC and fluorescence image indicating the subcellular localization of SPE-56::GFP in spermatids isolated from *spe-56(fed113)* males. Arrows mark punctate GFP signals corresponding to the localization of SPE-56. **(B)** Representative DIC (*a*, *c*) and immunofluorescence (*b*, *d*) images showing MSP localization using anti-MSP antibodies (red) in pronase-activated spermatozoa from *spe-56(fed113)* males and *spe-56(fed116)* null mutant males. DNA was stained with DAPI (blue). Arrows indicate MSP in the pseudopod. **(C)** DIC *(d,h)*, and immunofluorescence colocalization of SPE-56 and MOs in **(a–d)** *spe-56(fed113)* spermatids and **(e–h)** spermatozoa. Anti-GFP staining (green) marks SPE-56::GFP; anti-MO staining (red) labels MOs. Arrows indicate subcellular regions of SPE-56 localization and colocalization with MOs.

To determine the subcellular localization of SPE-56, we generated an integrated C-terminal GFP-tagged SPE-56 fusion allele, *fed113*, via CRISPR/Cas9. This allele is expressed from the endogenous *spe-56* locus under its native *spe-56* promoter. Homozygous *spe-56(fed113)* animals were phenotypically indistinguishable from wild-type animals with respect to self- and cross-fertility and sperm activation (Fig. 5A). Fluorescence microscopy revealed that SPE-56::GFP was expressed in both sexes in sperm. Punctate GFP signals were observed along the PM of the cell body resembling MO-associated sites under both in vivo and in vitro activation. Additionally, a faint GFP signal was detected in the cytosol of the cell body and pseudopod.

To confirm the molecular presence of SPE-56 in sperm, we conducted a Western blot analysis using an anti-GFP monoclonal antibody in sperm isolated from *spe-56 (fed113)* males. Given the low expression level of SPE-56 in the wild-type (Fig. 1D), we utilized the temperature-sensitive *fem-3(q96)* MOG (masculinization of germline) genetic background^68^, which is known to produce more sperm than the N2 wild type at 25 °C. Notably, Western blot analysis of the sperm lysates confirmed the presence of the SPE-56::GFP fusion protein at the cellular level (Fig. S5).

An immunofluorescence analysis using monoclonal anti-MSP antibodies in pronase-activated spermatozoa isolated from *spe-56(fed113)* or *spe-56(fed116)* deletion null mutants revealed the presence of MSP in the pseudopods of both strains, indicating cell polarization. However, MSP levels were lower in *spe-56(fed116)* null mutants than in the *spe-56(fed113)* control group (Fig. 5B, Fig. S4C), which is consistent with the shorter pseudopods of the mutant sperm (Fig. 3A).

To determine the spatial association of SPE-56 with MOs, we further conducted immunofluorescence colocalization experiments using anti-GFP and anti-MO antibodies in sperm isolated from *spe-56 (fed113)* males. Noteworthy, SPE-56::GFP localized predominantly to MO-positive puncta adjacent to the PM of the cell body (Fig. 5C, a–d). No detectable immunofluorescence was identified in the PM of the pseudopod (Fig. 5C, e-h).

These data indicate that in spermatozoa, SPE-56 is localized primarily to the membranes of fused MOs, which is consistent with its role in MO–PM fusion (Fig. 5C, e-h). SPE-56 functions as a key effector in the final maturation steps that render spermatozoa competent for fertilization.

### SPE-56 is a taxonomically restricted single-pass transmembrane protein with an intrinsically disordered C-terminus

To investigate the molecular features and potential functions of SPE-56, we employed standard bioinformatic methods for in silico analyses. BLAST searches identified conserved homologs in several *Caenorhabditis* species, additional members of the order *Rhabditida* (e.g., *Pristionchus pacificus*), and parasitic nematodes of the order *Spirurida* (e.g., *Brugia malayi*). No homologs were detected outside of the phylum *Nematoda*, indicating that SPE-56 is a taxonomically restricted, lineage-specific protein (Fig. S2).

**Fig. 6:**
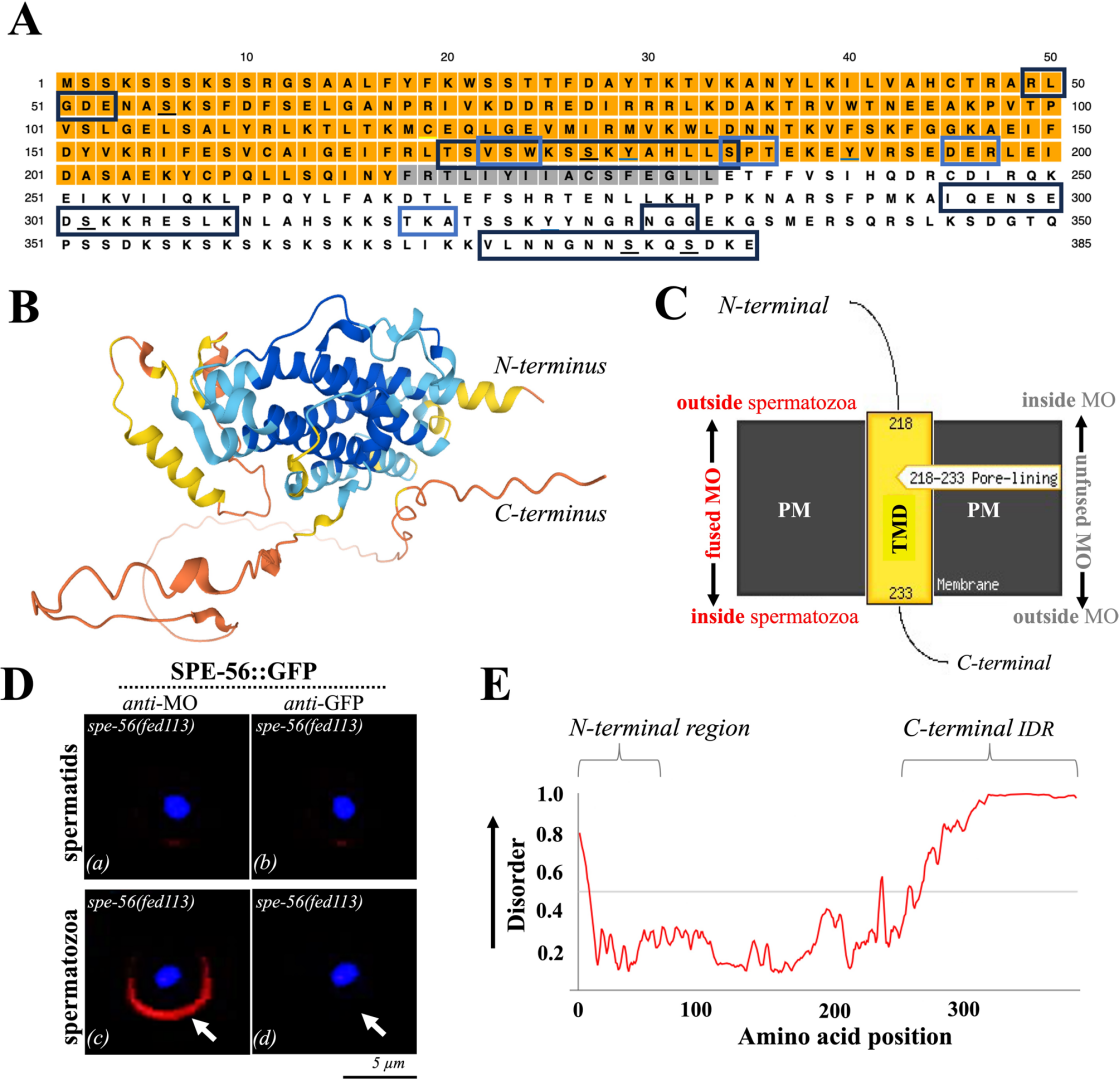
Bioinformatic and structural analysis of SPE-56. **(A)** Amino acid sequence of SPE-56 highlighting predicted domains via *PSIPRED* and post-translational modification sites using *PTMD 2.0*^70^. Predicted Serine (S) phosphorylation sites are underlined in black (residues 56, 177, 302, 379 and 382), the corresponding motifs are indicated by the black squares. Threonine (Y) phosphorylation sites are underlined in blue (residues 179, 190, and 325), the corresponding motifs are indicated by the blue squares. A palmitoylation site (C219) is underlined yellow. The transmembrane domain residues (218–233) are depicted on gray background, the amino acids of the C-terminal IDR are shown on white background, and the (predominantly) ordered region is highlighted in orange. **(B)** *AlphaFold* 3D structure of SPE-56^110^. The color scale denotes the confidence of prediction: blue (very high), light blue (high), yellow (low), and red (very low). **(C)** PSIPRED^69^ predicted transmembrane topology of SPE-56, showing its putative position in the plasma membrane (PM, green). The transmembrane domain (TMD) is shown in yellow; pore-lining residues (succession numbers) are indicated at the extracellular or cytosolic sides, which correspond to the intra- or extra-MO sides before fusion, respectively. **(D)** Immunofluorescence of non-permeabilized (see methods section) *SPE-56*::*GFP* spermatids ***(a***,*** b)*** and spermatozoa ***(c***,*** d)*** revealing the absence of the GFP-labeled IDR at the extracellular site of fusion pores confirming cytosolic orientation of the SPE-56 C-terminus (compare Fig. 5). Arrows indicate fused MO membranes (red) integrated in the PM. Nuclei are stained with DAPI (blue). **(E)** *AIUPred*^112^ profile of the SPE-56 sequence, indicating a high degree of disorder in the C-terminal region. Scores above 0.5 indicate disordered regions *(Erdős and Dosztányi*,* 2024).*

PSIPRED^69^ and PTMD 2.0^70^ analysis suggests that the SPE-56 sequence contains conserved serine/threonine phosphorylation sites and a cysteine palmitoylation site (Fig. 6A). Moreover, the serine/lysine (SK)-rich motifs at both termini, and particularly the N-terminal region (residues 2–15) show homology to serine-arginine/arginine-serine (SR/RS) domains of multifunctional nucleocapsid (N) proteins, such as those found in coronaviruses. These domains are known to mediate protein-protein and/or protein–RNA interactions^71,72^. Remarkably, the SPE-56 sequence lacks any known functional domains (Fig. 6A, C), however, it possesses a single-pass transmembrane domain (residues 218–233) and known tertiary folding structures, such as α-helices, β-pleated sheets, and random coils (Fig. 6B). No N-terminal signal peptide features have been identified^73^. Consistent with our colocalization data (Fig. 5) and predictions of transmembrane topology suggesting a cytosolic C-terminal IDR (Fig. 6C, E), SPE-56 primarily localizes to the membranes of MOs or fused MOs. To determine the precise location of the C-terminus, whether it is situated on the cytosolic side of the fused MO or extracellularly, a comparison of immunofluorescence colocalization signals was made in permeabilized (Fig. 5C) *versus* nonpermeabilized (Fig. 6D) spermatids and Pronase-activated spermatozoa isolated from *spe-56(fed113)* males expressing SPE-56::GFP. As expected, the nonpermeabilized spermatids exhibited no fluorescence signals for either anti-MO or anti-GFP (Fig. 6D a, b) antibodies, while nonpermeabilized spermatozoa exhibited robust anti-MO fluorescence along the outer PM of the cell body, indicating MOs fused with the PM (Fig. 6D, c). Strikingly, anti-GFP fluorescence signals corresponding to the C-terminus were completely absent (Fig. 6D, d), suggesting that this domain resides on the cytosolic side of the PM. Structural predictions further revealed that the cytosolic C-terminal region of SPE-56 is intrinsically disordered (Fig. 6E), enriched in S and K residues (Fig. 6A), and likely extends into the cytosol (Fig. 6C, D).

In summary, SPE-56 is a taxonomically restricted, single-pass transmembrane protein that appears to remain localized to the MO and does not disperse to the entire PM or pseudopod upon fusion, unlike other proteins such as SPE-9^11^. SPE-56 contains multiple potential regulatory interaction sites and features a large, intrinsically disordered C-terminal region exposed to the cytosol (Fig. 6), which is critical for its functional role in sperm activation.

### Deletion of the whole IDR disrupts SPE-56 function at all temperatures

**Fig. 7:**
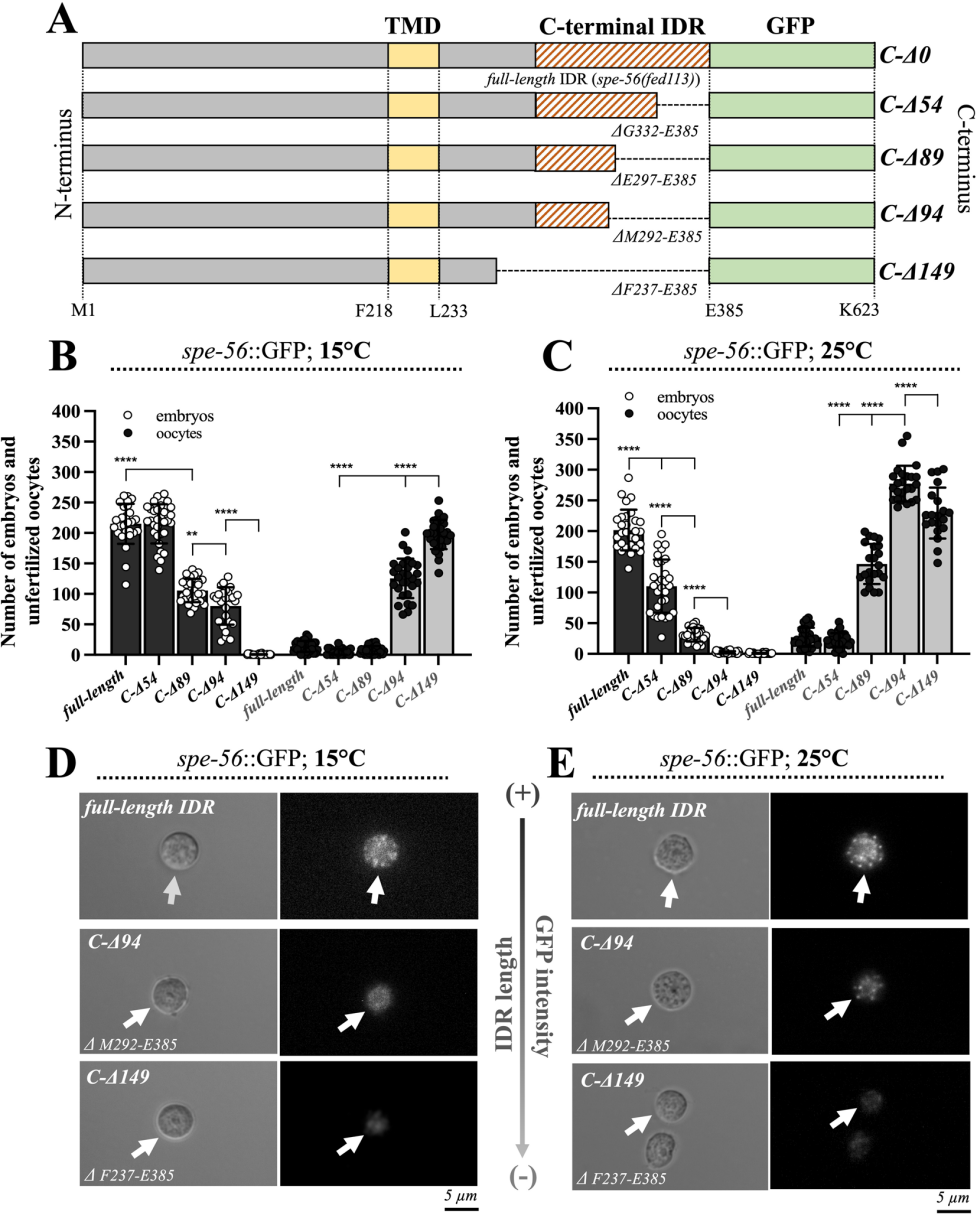
Temperature-dependent fertility of *spe-56* mutant sperm with C-terminal IDR deletions. **(A)** Schematic representation of CRISPR/Cas9-engineered C-terminal deletion alleles (C-Δ) in the *spe-56*::*GFP (allele fed113)* background. Numbers denote amino acid positions flanking the transmembrane domain (TMD), the intrinsically disordered region (IDR), the C-terminal GFP tag, and the respective deletion sites. **(B**,** C)** Quantification of embryos (black bars) and unfertilized oocytes (gray bars) laid by *spe-56*::*GFP* hermaphrodites harboring full-length or truncated (C-Δ) IDRs at 15 °C ***(B)*** and 25 °C ***(C)***. The values represent the means (± SD) of *N* = 3 independent experiments involving *n* ≥ 10 animals per trial. *p < 0.0332; **p < 0.0021; ***p < 0.0002; ****p < 0.0001 (Ordinary one-way ANOVA). **(D**,** E)** Differential interference contrast (DIC) and fluorescence microscopy images of single *spe-56(fed113)* spermatids carrying SPE-56::GFP with either the full-length C-terminal IDR or CRISPR/Cas9-engineered IDR deletion variants (*C-Δ94* or *C-Δ149*), grown at 15 °C **(D)** or 25 °C **(E)**. White arrows indicate spermatids. The gray long arrow between the images indicates the relative loss of GFP fluorescence with increasing IDR deletion.

While structured domains and short linear motifs typically mediate protein–protein interactions, intrinsically disordered regions (IDRs) can facilitate alternative interaction modes^74,75^. To evaluate the functional relevance of the C-terminal SPE-56 IDR, we created stepwise CRISPR/Cas9-mediated deletions (C-Δ) of 54, 89, 94, and 149 amino acids within the IDR sequence (Fig. 7A). Interestingly, these deletions caused in a progressive, temperature-sensitive decline in fertility in C-Δ hermaphrodites at both 15 °C and 25 °C (Fig. 7B) compared with *spe-56(fed113)* control animals carrying the full-length IDR. The C-Δ89 deletion mutant presented a significant reduction in embryo production, accompanied by an increase in unfertilized oocytes at 25 °C compared with 15 °C, whereas the C-Δ94 mutant was nearly completely sterile at 25 °C though it retained significant fertility at 15 °C (Fig. 7B, C). Additionally, fluorescence imaging revealed a gradual, IDR length-dependent decrease in the presence of SPE-56::GFP in spermatids, which was comparable at 15 °C and 25 °C (Fig. 7D, E; Fig. S3).

Mating assays involving temperature shifts from 15 °C to 25 °C prior to mating, using *spe-56(C-94)* mutant males and *fog-2(q71)* spermless hermaphrodites, indicate that temperature affects mutant spermatid quality, and consequently, fertility. The reduced ability of *spe-56(C-Δ94)* mutant spermatozoa to fertilize at 15 °C diminished further when meiotic differentiation occurred at 25 °C (Fig. S6B). The number of spermatids produced was unaltered (Fig. S6A).

These findings suggest that progressive truncation of the C-terminal IDR affects SPE-56 function in a temperature-sensitive manner. However, it remains unclear whether the deletions disrupt discrete functional motifs within the IDR or instead alter MO–PM fusion through changes in IDR length–dependent physiochemical properties.

## Discussion

The nematode *Caenorhabditis elegans*, while morphologically simple, presents a complex regulatory network governing sperm development and function. In this study, we identified *F56D5.2* as a previously uncharacterized gene essential for sperm activation. Based on its specific expression pattern revealed by RNA tomography^76^, its mutant phenotypes, and the established nomenclature^77^, we named this gene *spe-56*.

Functional analysis of *spe-56* deficiency and *null* alleles revealed a pronounced fertility defect linked to impaired sperm activation. Specifically, *spe-56* mutant sperm fail to initiate membranous organelle (MO) fusion with the plasma membrane (PM) and exhibit improper pseudopod extension, resulting in complete sterility and the production of unfertilized oocytes. These phenotypes were observed in both hermaphrodites and males, indicating a sperm-intrinsic defect rather than a defect in oocyte function^77,78^. These findings suggest that SPE-56 functions as a critical effector of sperm activation.

In *C. elegans*, male-derived sperm are typically transferred to the spermatheca, where they displace hermaphrodite sperm to gain a competitive fertilization advantage^79^. Although *spe-56*–deficient male sperm are successfully transferred to spermless *fog-2(q71)* hermaphrodites, they fail to persist within the spermatheca and are unable to outcompete wild-type N2 hermaphrodite sperm (Fig. 2A). *MitoTracker* staining^54,55^ indicates that *spe-56(t1791)* male sperm fail to maintain long-term residency in the spermatheca of mated hermaphrodites. These findings are consistent with successful production and transfer, but reduced motility due to the stubby, immotile pseudopods observed in both sexes (Figs. 3 and 4). The activation phenotype was validated in vitro using multiple activation protocols (Fig. S1), which confirmed the robustness of the in vivo observations.

Interestingly, the activation defects observed in *spe-56* mutants carrying the alleles *t1791* or *null* closely resemble those reported for *fer-1(hc1ts)-*deficient animals. FER-1 is a Ferlin family member which functions downstream of SPE-6 and SPE-4 within the canonical SPE-8 sperm activation pathway. It colocalizes with MOs during spermiogenesis, and has been demonstrated to be essential for MO–PM fusion and pseudopod extension - characteristics that are very similar to those of SPE-56^46^. These proteins likely exert their function in a downstream sub-branch of both activation pathways SPE-8^32^^[,60^^80^, and TRY-5^45^^[,81^. Mutations in these proteins result in activation failure and, consequently, infertility.

Recently, we identified the novel gene *spe-60*, which encodes a tau-tubulin kinase that is required for both meiotic differentiation of primary spermatocytes and normal pseudopod extension in spermatids. SPE-60 colocalizes with MOs. Unlike SPE-56, SPE-60-deficient sperm fail to form completely extended pseudopods but show normal MO fusion^82^. These findings imply that MO fusion is necessary for fertilization^11,39^, yet insufficient for full pseudopod extension. Thus, key functional MO-associated proteins such as FER-1, SPE-60, and SPE-56 are likely required for normal pseudopod morphogenesis, including the full-length extension. Moreover, STRING analysis^83^ suggested a possible interaction between SPE-56 and SPE-60.

Remarkably, the SPE-56 function appeared to be closely associated with the state of its C-terminal IDR. In literature, the apparent lack of IDR conservation across a great variety of proteomes has sometimes been interpreted as indicative of minimal functional relevance. Nevertheless, accumulating evidence - including from this study - has demonstrated that IDRs play essential roles in diverse molecular and cellular processes^84–86^. In line with this, our CRISPR/Cas-based editing approach revealed that SPE-56 deletion mutants (*C-Δ*) lacking 54, 89, 94, or 149 amino acids within the protein C-terminus (Fig. 7A) resulted in a gradual temperature-responsive loss of fertility (Fig. 7B, C). Additionally, it is important to note that the number of spermatids produced by *spe-56(t1791)* mutant males through meiotic differentiation was similar to that of N2 wild types at 15 °C and 25 °C (Fig. S6A). Given that spermatogenesis is generally a temperature-dependent process, these results suggest that the IDR function of SPE-56 in spermatozoa depends on length rather than on temperature (Fig. 7D, E; Fig. S3 A, B). Furthermore, it is implied that meiotic spermatid output remains quantitatively unaltered (i.e., the number of spermatids, Fig. S6A) and that the IDR may contribute in a length-dependent manner to the segregation of SPE-56 and other spermatogenic proteins (e.g., MSP) through the final stage of meiotic differentiation (Fig. 7D and E, Fig. S3). However, it remains to be elucidated whether SPE-56 has a discrete function in meiosis.

In light of the established understanding that both processes, MO fusion and normal pseudopod extension, are induced by the same pathway downstream of SPE-6, and in accordance with our hypothesis that SPE-56 is an MO-associated protein with a C-terminal cytosolic presence, it is reasonable to consider the possibility of its interaction with cytosolic and/or MO-associated proteins, such as MSP, that is released by MOs to enable normal pseudopod function and morphology. The stubby pseudopods (Fig. 3A) are consistent with the lower presence of MSP in *spe-56(null)* mutants (Fig. 5B; Fig. S4C). In order to identify the key proteins that link the two processes, modifier screens using EMS mutagenesis on several temperature-sensitive C-terminal deletion mutants of *spe-56* could be an appropriate method.

An emerging paradigm suggests that IDR conservation may be encoded at the level of physicochemical features, such as charge, polarity, and hydropathy, rather than amino acid identity^87–89^. However, IDRs are typically enriched in residues that promote disorder, including charged and polar amino acids^90^. As shown in Fig. 6A, the IDR of SPE-56 is dominated by polar amino acids, a compositional profile that resembles the IDR of SPE-18 - another *C. elegans* sperm protein essential for spermatocyte development in combination with SPE-6 - facilitating chromosome segregation and MSP partitioning^27,62^. Both *null* mutants of *spe-18* and *spe-56* are sterile due to defective spermatogenesis. However, despite their putative influence on MSP assembly or segregation, their particular roles in sperm maturation are distinct. While SPE-18 is required during early meiotic differentiation, SPE-56 acts later, specifically during spermiogenesis. Although the IDR of SPE-18 is essential for its function, this function also depends on the presence of ordered protein regions along the SPE-18 sequence. Consistent with these observations, our deletion analysis demonstrated that the C-terminal IDR is an indispensable component of SPE-56 functionality (Fig. 7 and S3). However, the potential contributions of other protein domains, such as the TMD and/or the N-terminus, to the subcellular location and normal function of SPE-56 cannot be disregarded (Fig. 6C).

Overall, these findings support the broader concept that IDRs, despite their low sequence conservation, are indispensable for specific biological functions in *C. elegans* and other species.

The application of immunofluorescence analysis and PSI-PRED structural prediction led to the classification of SPE-56 as a single-pass transmembrane protein with an intrinsically disordered region (IDR) located on the cytosolic side of the membrane (Figs. 5C and 6C and D). Nevertheless, additional evidence for the proposed topology would be provided by a N-terminal GFP-tagged SPE-56. Notably, 63% of the predicted IDRs in single-pass membrane proteins are localized to the cytosolic side, a proportion that increases to 81% for multipass membrane proteins. Topological features are often predicted via the “positive-inside” rule, which posits that positively charged residues flanking transmembrane domains preferentially orient toward the cytoplasm, where they frequently serve as hubs for signaling interactions^90,91^. Consistent with this, as well as with the localization of MOs within the sperm cytosol prior to PM fusion, the C-terminus of SPE-56 - enriched in lysine (K) and serine (S) residues - forms a highly charged sequence outside the fused MO, though not outside the sperm cell (Fig. 6D).

In addition to their structural flexibility, IDRs are also increasingly recognized for their function in phase separation and subcellular compartmentalization^92–94^. In *C. elegans*, the IDR-containing paternal-effect gene *spe-11* encodes a sperm-derived protein required in oocytes for embryonic development^40,95^ driving liquid–liquid phase separation (LLPS), a property essential for granule formation during early embryogenesis^96^. Similarly, paternal epigenetic inheritance (PEI) granules, which contain the Argonaute protein WAGO-3, represent a class of germline LLPS assemblies critical for normal sperm development. Deletion of IDRs in PEI granule proteins has been shown to disrupt WAGO-3 incorporation into sperm, suggesting that IDRs are involved in cargo recruitment processes^28^. Although we did not identify phenotypes involving the recruitment or organization of additional sperm components necessary for paternal fertility during early spermatogenesis, except for a potential effect on MSP (Fig. 3A*d*, *f*, Fig. 5B), our results suggest that the IDR of SPE-56 may influence its segregation during the final stage of meiotic differentiation (Fig. 7). Nevertheless, the function of SPE-56 affecting meiosis remains ambiguous since the number of spermatids produced by *spe-56* mutants is similar to that of wild-type at all temperatures (Fig. S6A). In this context, however, it should be noted that temperature may affect the function of the SPE-56 IDR during the final stage of meiotic differentiation (Fig. S6B). This could involve interaction with MSP and/or other spermatogenic proteins, through posttranslational modifications such as phosphorylation and palmitoylation, which are characteristic of transmembrane protein interactions (Fig. 6A and Fig. S2).

Phosphorylation is a key posttranslational modification in signal transduction, and the amino acid sequence of SPE-56 is notably enriched in predicted phosphorylation sites at serine (S) and threonine (T) residues (Fig. 6A). Proteins with such features are typically targets for regulatory enzymes such as kinases and phosphatases, which are disproportionately abundant in sperm^97,98^. Additionally, SPE-56 contains a predicted palmitoylation site at Cys-119 (Fig. 6A), which may be necessary for facilitating membrane-to-membrane contact during MO fusion. Palmitoylation is described as a posttranslational modification known to facilitate protein–membrane interactions and has critical functions in modulating membrane targeting and vesicle trafficking^99–101^ which are hallmark-features of the acrosome reaction in the mammalian sperm. Knockout of the *Zdhhc19* gene, which encodes a palmitoyl transferase, results in reduced sperm motility and abnormal membrane fusion events, highlighting the importance of palmitoylation in maintaining sperm membrane integrity^102,103^.

In *C. elegans*, the palmitoyl transferase SPE-10 is a DHHC-CRD zinc-finger transmembrane protein required for fibrous body (FB)-MO assembly during spermatogenesis. SPE-10 localizes to the ER/Golgi membrane, and its loss leads to disrupted FB-MO formation and sperm sterility^42,104^. In contrast, SPE-56 deficient animals display no abnormalities in spermatocyte morphology or early development. Instead, fertility defects arise specifically during spermiogenesis because of incomplete sperm activation. However, SPE-56 palmitoylation by SPE-10 during early spermatogenesis cannot be ruled out. Furthermore, SPE-21, a DHHC-CRD-type zinc finger motif palmitoyl transferase localized to the MOs, is required for proper spermatid activation, including MO-PM fusion and pseudopod formation. Putative *spe-21(null)* mutant worms are severely sub-fertile and resemble to a certain degree the phenotypes of s*pe-56(null)* mutants^105^. Consequently, it can be posited that SPE-21 and SPE-56 may be members of a common pathway, given their shared subcellular location and the observation that defects in these proteins cause similar phenotypes.

In human sperm, the palmitoylated cysteine string protein (CSP) is associated with the PM, playing a central role in acrosomal exocytosis by mediating trans-SNARE complex assembly between the acrosome and the PM^106^. Analogously, SPE-56 is a single-pass transmembrane protein localized to the MO membrane (Figs. 5 and 6) and is essential for MO–plasma membrane fusion (Fig. 4A, B). On the basis of these features, we propose that the IDR of SPE-56 is required for membrane contact at the MO–PM interface to initiate and/or facilitate membrane destabilization and fusion. This contact likely represents a critical early step in the fusion process that is disrupted in the absence of functional SPE-56.

Taken together, our findings provide new insights into the molecular mechanisms underlying spermiogenesis and establish SPE-56 as a critical regulator of MO fusion and pseudopod morphogenesis at the interface between the PM and MOs. Most probably, SPE-56 functions alongside other SPE proteins - such as FER-1 and SPE-60, which operate downstream of SPE-8 and SPE-6 - to facilitate similar processes. Our results also support the emerging hypothesis that MO-associated proteins, rather than the process of MO fusion per se, are required to establish normal pseudopod morphology and function. Moreover, elucidating the dynamics at the MO‒PM interface may shed light on indispensable steps essential for sperm fertility across species. Similar to the acrosome reaction in mammals or similar events (e.g. endo- and exocytosis), mechanisms that mediate membrane remodeling and fusion may rely on transmembrane proteins enriched in IDRs. These findings support the idea that such fusion mechanisms are most likely evolutionarily conserved across species and rely on conserved protein functions rather than on the specific sequence identities of the proteins involved. In the particular context of this study, SPE-56 has the potential to contribute to the development of a comprehensive framework for understanding the specific structural transitions necessary for fertility, which are coordinately regulated by disordered protein domains.

## Methods

### *C. elegans* strains and culture conditions

All *Caenorhabditis elegans* strains were maintained under standard laboratory conditions on nematode growth medium (NGM) agar plates seeded with *Escherichia coli* OP50 at 20 °C, following established protocols^29^. The canonical wild-type strain N2 (Bristol) served as the reference background for all comparative analyses. The mutant and transgenic strains used in this study were obtained from the *Caenorhabditis* Genetics Center (CGC) and the National Bioresource Project (NBRP), unless otherwise specified. The strains included VC4606 *F56D5.2(gk5676)IV*, BA984 *spe-6(hc163)*, CB4108 *fog-2(q71)V*, JK1074 *fem-3(q96)IV*, JJ1271 *glo-1(zu391)X*, and EG5801 *oxSi87II. oxSi87* contains [*peel-1p*::N-terminal 12 amino acids of PEEL-1::GFP::*peel-1* 3’UTR + Cbr-*unc-119*(+)] II to drive GFP expression in the spermatogenic germline^41^. The alleles *F56D5.2(t1791)* and VC4606 *F56D5.2(gk5676)* were generated by the *Moerman* lab (UBC Vancouver, Canada) in an effort to obtain *null* mutations in all *C. elegans* genes^47^. This strain contains a marker cassette carrying *[loxP+Pmyo-2*::*GFP*::*unc-54 3’UTR + Prsp-27*::*neoR*::*unc54 3’UTR + locP]/+IV.*

The following CRISPR/Cas9-engineered strains/alleles of *spe-56* were designed by us und manufactured by SUNY BIOTECH Co., Ltd.: PHX3895 (syb3895): C-terminal GFP-tagged translational fusion of *spe-56*, designated as *fed113;* PHX4469 (syb4469): GFP-tagged translational fusion and functional mutation affecting the entire genomic locus (deletion *null* allele) designated as *fed116*. The *spe-56* genomic sequence was replaced by GFP under the pharynx-specific *myo-2* promoter (*myo-2*p::GFP) and maintained as heterozygous *fed116*/+ or balanced with the *n*T1[qIs51]IV,+/*n*T1[qIs51]V (reciprocal translocation); PHX4786 (syb4786): C-terminal GFP-tagged translational fusion with the *spe-56* IDR-deletion allele (−543 bp; −149aa) designated as *C-Δ149*, *n*T1[qIs51]IV,+/*n*T1[qIs51]V balanced; PHX6819 (syb 6819): C-terminal GFP-tagged translational fusion with the *spe-56* IDR-deletion allele (−321 bp; −94aa) designated as *C-Δ94*; PHX5208 (syb5208): C-terminal GFP-tagged translational fusion with the *spe-56* IDR-deletion allele (−336 bp; −89aa) designated as *C-Δ89;* PHX4012 (syb4012): C-terminal GFP-tagged translational fusion with the *spe-56* IDR-deletion allele (−216 bp; −54aa) designated as *C-Δ54*.

All the mutant strains were backcrossed at least four times to the N2 wild-type background (ratio: 2 hermaphrodites with 10 males) to eliminate interfering background mutations and ensure phenotypic consistency. It is noteworthy that backcrossing with the deletion null allele gk5676 (VC4606) was unsuccessful after several attempts. However, its phenotype was consistent with those exhibited by *fed116* and other (backcrossed) *spe-56* deletion alleles.

Genotypes of transgenic *spe-56* strains were confirmed by PCR amplification using the pri-mers 5’-cctcgaaattgagaactgcaagacc-3‘ and 5’-cagtccgcgaaaattctatgagcc-3’ for all alleles except *fed140*, where the primers 5’-actcaagaaccttgctcacagc-3’ and 5’-cagtccgcgaaaattctatgagcc-3’ were used. All sterile *spe-56* mutant lines were maintained via the reciprocal translocation *n*T1[qIs51]*(IV; V)*, which carries a *myo-2p*::GFP marker enabling the selection of heterozygotes by pharyngeal fluorescence. Double mutant strains were generated via conventional genetic crosses and confirmed via PCR genotyping followed by sequencing of the relevant genomic regions. All experiments were conducted on synchronized *C. elegans* populations grown under *ad libitum* feeding at 20 °C to ensure developmental consistency across genotypes.

### Fertility assays

The evaluation of male fertility was conducted by culturing synchronized L4-stage males for 48 h on 35-mm NGM agar plates under conditions of *ad libitum* feeding at 20 °C as described by *Gottschling and Döring*^82^. Mating assays were performed using six adult males and two virgin *fog-2(q71)* hermaphrodites per plate. After an overnight mating period, the inseminated hermaphrodites were isolated and transferred daily to fresh plates until they ceased laying eggs. Their progeny were counted at the L4 or young adult stage (48 h post-hatching). Control experiments were conducted with wild-type N2 males. Control males were generated via heat shock on five L4 hermaphrodite larvae, which were incubated for five hours at 30 °C with *ad libitum* feeding on 35 mm plates seeded with *E. coli* OP50. This was followed by a recovery period at 20 °C. Male progeny were then selected and maintained by crossing them with hermaphrodites. Mutant males were generated by applying the standard heat shock treatment (five hours at 30 °C) to *n*T1-balanced L4 mutant hermaphrodites expressing an *n*T1-integrated pharynx-GFP. These males were crossed with hermaphrodites of the same genotype. Unbalanced mutant males (with no pharynx-GFP) were selected and used for the experiments. For the hermaphrodite self-fertility assays, *spe-56(null)* L4 larvae were transferred individually to 35 mm NGM agar plates. They were allowed to lay eggs at 20 °C under *ad libitum* feeding conditions. Adults were transferred daily to new plates for brood size quantification.

Sperm transfer was assayed by mating *spe-56(t1791)* mutant or N2 wild-type males with *fog-2(q71)* hermaphrodites. The males were placed in 500 µL of 10 µM *MitoTracker Red CMXRos*, which stains the mitochondria of living cells^54,55^. To label the sperm, these were incubated for two hours in the dark in small glass dishes. Then, the males were removed from the solution with a mouth pipette and placed onto a 60 mm agar plate seeded with an *E. coli* OP50 culture. Thirty minutes later, only active worms were chosen to mate with isolated L4 hermaphrodites overnight. The experiment was conducted with *fog-2(q71)* hermaphrodites mated with *spe-56(t1791)* or N2 wild-type males in a ratio of 1:8. We visualized post-mating transferred sperm using differential interference contrast (DIC) and fluorescence microscopy with an AxioImager M2 microscope (Zeiss)^82^.

### Sperm isolation and in vitro activation

Sperm activation assays were performed using celibate males and hermaphrodites with wild-type or mutant genetic backgrounds cultured under *ad libitum* feeding conditions. L4-stage worms were transferred to 35 mm NGM agar plates and incubated at 20 °C for 48 h. For sperm isolation, adult worms were transferred to a 4.5 µL droplet of sperm medium (SM buffer; 50 mM HEPES, 45 mM NaCl, 1 mM MgSO₄, 25 mM KCl, 5 mM CaCl₂, 10 mM dextrose; pH 7.8)^65^ placed on a glass microscope slide (Thermo Scientific) pre-marked with a hydrophobic PAP pen (DAKO). Coverslips (20 × 20 mm; Carl Roth) were then applied, and the samples were sealed with Vaseline. Spermatozoa were released by mechanically dissecting the gonads via two 25-gauge needles under a stereomicroscope. For in vitro activation of male-derived sperm, 0.5 µL of SM buffer containing either an ectopic in vitro activation trigger (e.g., pronase) or buffer alone (control) was added to the preparation. The samples were incubated for 30 min at 20 °C prior to imaging. Pronase (2 mg/mL in SM buffer) served as the standard activator unless otherwise indicated. Following activation, a 20 × 20 mm glass coverslip (Carl Roth GmbH) was gently placed over the sample and sealed with Vaseline to prevent evaporation during microscopy^82^.

Sperm numbers were scored individually via microscope on 10 worms, which were prepared as described above. Sperm activation was evaluated through the implementation of differential interference contrast (DIC) and fluorescence microscopy. The classification of individual sperm was based on cell morphology and the presence of MO fusion pores. Cells exhibiting a spherical morphology, devoid of pseudopod extension and MO fusion pores, were designated as nonactivated. Sperm that exhibited clearly visible fusion pores and motile, fully extended pseudopods—defined as projections at least equal in length to the cell body—were designated as activated^107^. Furthermore, spermatozoa exhibiting features such as MO fusion and pseudopods of a shorter length compared to the cell body (“stubby” phenotype), were also designated as activated.

### MO fusion assays

The assessment of MO fusion was conducted using a lipophilic membrane dye-based assay, as previously outlined in the literature^46^, with minor modifications, as defined by *Gottschling and Döring*^82^. In summary, isolated spermatids or in vitro-activated spermatozoa were incubated in sperm medium (SM buffer) containing 5 µg/mL FM 1–43 dye (obtained from Thermo Scientific) for a period of three minutes at room temperature. Subsequent to the process of incubation, the samples were mounted under a 20 × 20 mm glass coverslip and imaged immediately via a Zeiss laser scanning confocal microscope (LSM700) equipped with a 100-fold oil immersion objective and an AxioCam MRm camera (Zeiss). Successful MO fusion was determined by the presence of punctate FM 1–43 fluorescence localized along the PM. Cells exhibiting an absence of a membrane-associated FM 1–43 signal were designated as nonactivated.

### Quantification of sperm activation

Sperm activation was quantified in synchronized worm populations grown at 20 °C under *ad libitum* feeding conditions. To ensure the maintenance of male celibacy, L4-stage males were isolated from hermaphrodites and cultivated in individual cultures for a period of 48 h. Subsequently, the adult males were dissected as previously described to isolate spermatozoa.

The activation status of the sperm was then assessed by incubating isolated sperm in sperm medium (SM buffer) supplemented with 5 µg/mL FM1-43 (Thermo Scientific)^46,108^ for a period of three minutes. The presence of pseudopod extension and membrane-localized fusion pores was used as a combined indicator of successful activation. Cells exhibiting both of these features were designated as “activated,” whereas cells with a spherical morphology, devoid of pseudopods and fusion-associated fluorescence, were classified as “nonactivated.” The extent of spontaneous (premature) activation of male sperm was categorized according to established benchmarks^64^.

### Microscopy

High-resolution microscopy of spermatozoa was performed in both in vivo and in vitro settings via a Zeiss AxioImager M1 DIC and fluorescence microscope equipped with a 100-fold oil immersion objective and an AxioCam MRm camera (Zeiss) described by *Gottschling and Döring*^82^. To perform live imaging of sperm in whole worms, 10 males or hermaphrodites were anesthetized in 5 µL of 5 mM levamisole and mounted on 2% agarose pads prepared on standard glass microscope slides (Thermo Scientific). The immobilized worms were then imaged in vivo at 20 °C.

The imaging of isolated sperm cells was performed by first dissecting cells from adult males or hermaphrodites and subsequently suspending them in 4.5 µL of SM buffer, with or without prior in vitro activation. The samples were sealed under a 20 × 20 mm glass coverslip using Vaseline to prevent evaporation. The visualization of GFP-tagged fusion proteins was accomplished at 488 nm using the appropriate Zeiss filter sets. Image acquisition was performed via time-to-live software (Schnabel Lab, TU Braunschweig, Germany). The images were captured as.lurawave files and subsequently cropped, converted, and saved in a format suitable for publication, such as.jpg,.bmp, or.png.

The imaging of fixed immunostained sperm was conducted via a Zeiss AxioImager Z2 upright microscope coupled with an LSM 700 laser scanning confocal module at 488 nm for green (Alexa 488, Thermo Scientific), 555 nm for red (Alexa555, Thermo Scientific), and 405 nm for DAPI-stained nuclei. The images were saved as.czi files and subsequently cropped and converted into a format suitable for publication (e.g.,.jpg,.bmp, or.png) using ZEN imaging software (Zeiss).

### Immunostaining

Immunofluorescence staining of the isolated sperm was performed on poly-L-lysine-coated glass slides (Thermo Scientific) as described by *Gottschling* and *Döring*^82^. A hydrophobic barrier was drawn with a PAP pen (DAKO) to restrict the spread of the sample. Ten adult males were dissected within the marked area in 3 µL of sperm medium (SM buffer) to release spermatocytes, spermatids, and/or spermatozoa. The released cells were fixed by adding 3 µL of 4% paraformaldehyde (PFA) in SM buffer and incubated for 30 min at 20 °C. After fixation, the samples were washed with 20 µL of PBST (PBS supplemented with 0.01% Tween-20, Carl Roth GmbH) and permeabilized with 20 µL of PBS containing 0.5% Triton X-100 (Carl Roth GmbH) for five minutes. After a second PBST wash, the samples were blocked in PBST containing 0.5% BSA for 30 min at room temperature.

The primary antibody incubation was performed for one and a half hours at room temperature using rabbit anti-GFP (1:1000; GF28R, Thermo Scientific) mouse monoclonal anti-MO 1CB4 (1:1000) diluted in PBST with 0.5% BSA. After three 10-minute washes in PBST, the samples were incubated for 30 min with 1:500 dilutions of Alexa Fluor 488-conjugated anti-rabbit and Alexa Fluor 555-conjugated anti-mouse secondary antibodies (Thermo Scientific) in PBST + 0.5% BSA. The 1CB4 antibody was provided by Ralf Schnabel (Schnabel Lab, Developmental Genetics, TU Braunschweig, Germany). After the secondary incubation, the slides were washed three times for 10 min each in PBST and mounted with DAKO mounting medium supplemented with 2 ng/µL DAPI (Thermo Scientific) for nuclear counterstaining. Imaging was performed using a Zeiss AxioImager Z2 microscope equipped with an LSM 700 laser scanning confocal system, and image processing was conducted using ZEN software (Zeiss). The localization of the MSP was performed using mouse anti-MSP 4A5 antibodies (Developmental Studies Hybridoma Bank, University of Iowa) under the same conditions described above.

### Immunoprecipitation and Western blot

Synchronized *fem-3(q96)ts* single mutants and *spe-56(t1791)*,*fem-3(q96)* double mutants were grown at 15 °C until the early L4 stage. Then, they were incubated 24 h at 25 °C to induce the *fem-3(q96)-*specific MOG (masculinization of germline) phenotype. This resulted in continuous sperm production instead of the normal switch to oogenesis. Sperm cells were isolated from 100 adult animals (10 per step) using a Sterican/Braun needle (0.40 × 20 mm) and dissection in SM buffer at room temperature on a microscope slide (Epredia). Subsequently, the cells were transferred with a mouth pipette (self-construction) to and collected in low-binding 1.5-ml microtubes (Sarstedt). The samples were then stored at −20 °C. For analysis, the frozen sperm cells were resuspended in ice-cold RIPA lysis buffer (50 mM Tris-HCl, 150 mM NaCl, 1% Triton X-100, 0.5% Na-deoxycholate, 0.1% SDS, pH = 8.0, supplemented with *c*Omplete™ Mini EDTA-free protease inhibitor cocktail (Roche)) and sonicated (4 × 30 s). After centrifugation (20,000 × g for 15 min at 4 °C), the supernatant was collected for immunoprecipitation via GFP-Trap agarose beads (ChromoTek) according to the manufacturer’s protocol. The trap is based on anti-GFP nanobodies coupled to the beads, which bind GFP fusion proteins with high affinity. The supernatant containing the sperm proteins was incubated with 25 µL GFP-Trap agarose beads with end‒over-end rotation for 1 h at 4 °C, followed by three washes with buffer (10 mM Tris-Cl pH = 7.5, 150 mM NaCl, 0.05% Nonidet™ P40 Substitute, 0.5 mM EDTA, and 0.018% Na-azide). Elution of the bound proteins was performed by boiling the beads for 5 min at 95 °C in 80 µL of 2x Laemmli buffer (Thermo Fisher Scientific). Next, 25 µL bound fraction per lane was separated via SDS‒PAGE on a 4‒12% Bis‒Tris mini gel (iD‒PAGE, Eurogentec) in MOPS running buffer at 160 V. Protein fractions were transferred to a nitrocellulose membrane for 30 min at 100 V in Tris-Glycine running buffer (Bio-Rad, 20% EtOH). After blocking in 5% milk in PBS-T (0.01% Tween-20) for 1 h, the membrane was incubated with the primary anti-GFP antibody (1:1000) in 5% milk in PBS-T (0.01% Tween-20) overnight at 4 °C, followed by 3 × 15 min washes in PBS-T and a 1 h incubation with the secondary antibody (1:10,000) at room temperature, followed by a 3 × 15 min wash in PBS-T. Chemiluminescence detection was performed using Clarity ECL substrate (Bio-Rad).

### Bioinformatic analysis

General information about the genome and protein sequences was obtained from *Wormbase* (Version 2024; https://wormbase.org/). Disorder predictions were performed using AlphaFold (V. 2)^109^^[,110^, MobiDB (V. 6.2; https://mobidb.org)^111^, and AIUPred (V. 1.2.1; https://aiupred.elte.hu)^112^. The functional protein domains were predicted using PSIPRED (V. 2025; http://bioinf.cs.ucl.ac.uk/psipred/)69, posttranslational modifications via PTMD (V. 2; https://ptmd.biocuckoo.cn)^70^, and N-terminal signaling features using DeepSig (V. 2025; https://busca.biocomp.unibo.it/deepsig/)^73^. For sequence alignments, homologs were identified using the standard protein blast algorithm BLASTP (V. 2025; https://blast.ncbi.nlm.nih.gov/) and aligned with Clustal Omega (V. 2022; https://www.ebi.ac.uk/jdispatcher/msa/clustalo) at the UniProt database (V. 2020_01; https://www.uniprot.org/). Expression was analyzed using TomoSeq (V. 2026; http://celegans.tomoseq.genomes.nl/)^49^, WormSeq (V. 2026; https://37nyza-abbas-ghaddar.shinyapps.io/shiny_webpage/)^53^, the interactive visualizer tool of gene expression in the early *C. elegans* embryo (V. 2026; https://tintori.bio.unc.edu)^51^, and PAXDB 6 (V. 2026; https://pax-db.org/species/6239)^52^.

### Statistical analysis

Statistical analyses were performed using GraphPad Prism (10.4.2). To assess differences between groups, we employed an ordinary one-way ANOVA for multiple comparisons. The significance levels are as follows: *p < 0.0332, **p < 0.0021, ***p < 0.0002, and ****p < 0.0001. Unless otherwise stated, the data are presented as means ± standard deviations (SD). Sample sizes (n) refer to biologically independent replicates, and no data points were excluded unless otherwise stated. No corrections for multiple comparisons were applied. To ensure reproducibility, the data represent the means ± SDs of three independent experiments involving at least ten animals per trial.

## Supplementary Information

Below is the link to the electronic supplementary material.

Supplementary Material 1

## Data Availability

The raw data supporting the findings of this study are available from the corresponding author at any time upon request (E-mail: *gottschling@molprev.uni-kiel.de*). The processed data can be found within the manuscript or in the supplementary information files.
