# Correction: The intrinsically disordered protein SPE-56 is required for acrosomal-like exocytosis and fertility in *Caenorhabditis elegans*

**DOI:** 10.1038/s41598-026-63188-6

**Published:** 2026-07-21

**Authors:** Dieter-Christian Gottschling, Sarah Eiser, Frank Döring

**Affiliations:** Department of Molecular Prevention, University of Kiel, 24118 Kiel, Germany

Correction to: *Scientific Reports* 10.1038/s41598-026-47896-7, published online 11 April 2026

The original version of this Article contained errors.

Firstly, in the Results section, under the subheading ‘Deletion of the whole IDR disrupts SPE-56 function at all temperatures’, the term ‘amino acids’ was inadvertently replaced with “base pairs”.

As a result, where:

“To evaluate the functional relevance of the C-terminal SPE-56 IDR, we created stepwise CRISPR/Cas9-mediated deletions (C-Δ) of 54, 89, 94, and 149 base pairs within the IDR sequence (Fig. 7A).”

now reads:

“To evaluate the functional relevance of the C-terminal SPE-56 IDR, we created stepwise CRISPR/Cas9-mediated deletions (C-Δ) of 54, 89, 94, and 149 amino acids within the IDR sequence (Fig. 7A).”

In addition, Figure 1 contained a display error in panel C, where the micrometre scale bar was missing.

**Fig. 1:**
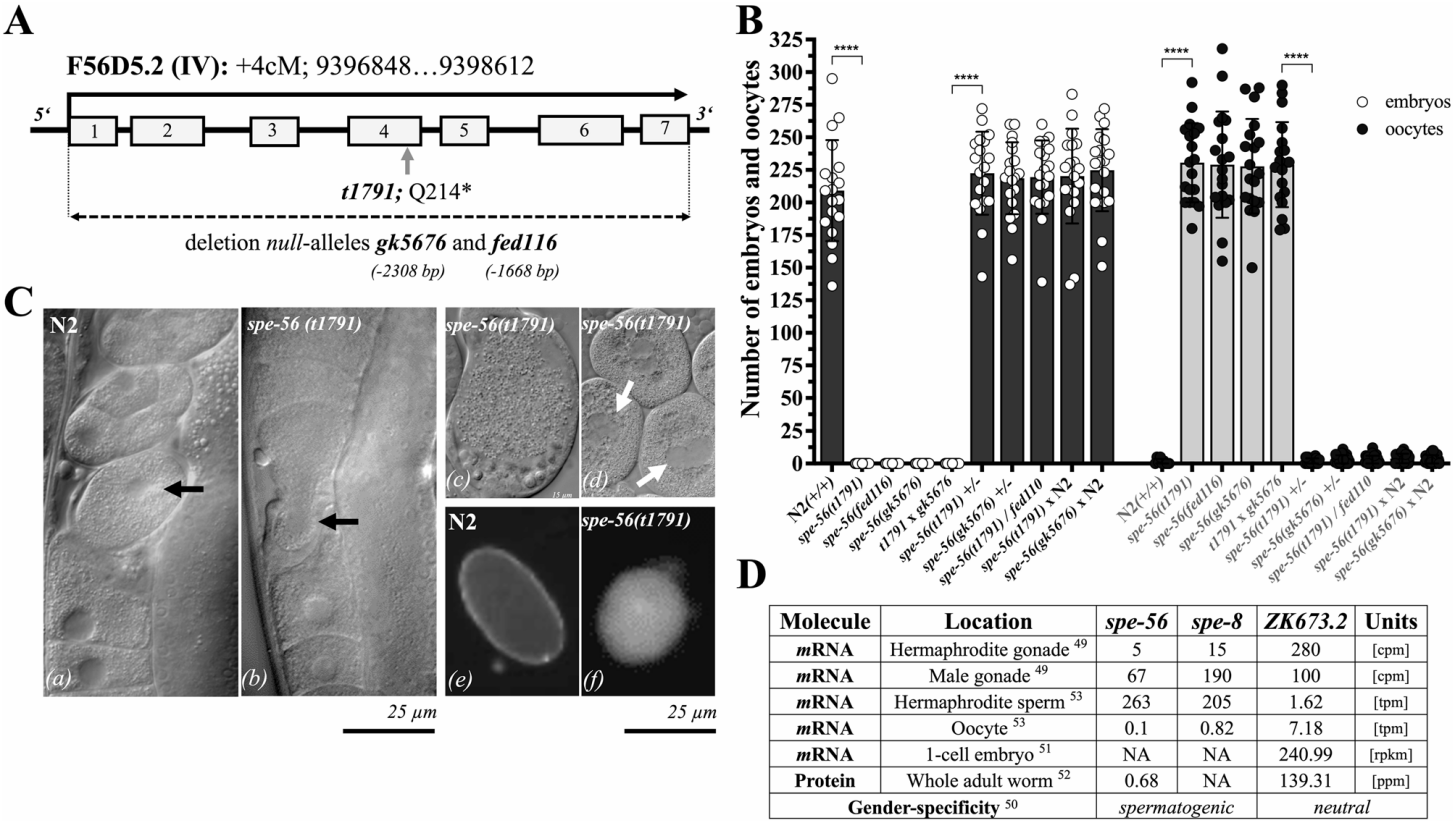
Structure and fertility phenotypes of *F56D5.2* mutant alleles. **(A)** Schematic of the *F56D5.2* gene structure showing the *t1791* allele (gray arrow), which results in a premature STOP (*) codon at Q214, and the CRISPR/Cas9-generated *null*-alleles *gk5676* and *fed116* encompassing the deletion of the entire *F56D5.2* genomic sequence. **(B)** Numbers of embryos and unfertilized oocytes produced by *F56D5.2* homozygous and heterozygous mutant hermaphrodites, including rescued and trans-heterozygous animals. The values represent the means (± SD) of *N* = 3 independent experiments involving *n* ≥ 10 animals per trial. *p < 0.0332; **p < 0.0021; ***p < 0.0002; ****p < 0.0001 (Ordinary one-way ANOVA). **(C)** Differential interference contrast (DIC) and fluorescence images depicting the uterus of a wild-type (N2) hermaphrodite harboring fertilized oocytes and early embryos ***(a)*** compared with the uterus of a *t1791* mutant hermaphrodite filled with unfertilized ***(b)*** and endomitotic oocytes (EMOs) ***(c***,*** d)***. Oocytes are indicated by black arrows. White arrows indicate multiple endomitotic nuclei. *Trypan-blue* staining reveals abnormal membrane permeability of EMOs in *t1791* mutants ***(f)*** compared to fertilized N2 wild-type oocytes enclosed by an eggshell ***(e)***. **(D)** Expression profiles of *spe-56*, *spe-8*, and *ZK673.2* based on transcriptomic and proteomic data. *spe-56 m*RNA is primarily found in sperm. The units are “counts per million” (cpm), “transcripts per million” (tpm), “reads per kilobase million” (rpkm), and “parts per million” (ppm).

And lastly, the amino acids G332, E297 and M292 were incorrectly captured as “G331”, “Q296” and “P292” in Figure 7 panel A.

**Fig. 7:**
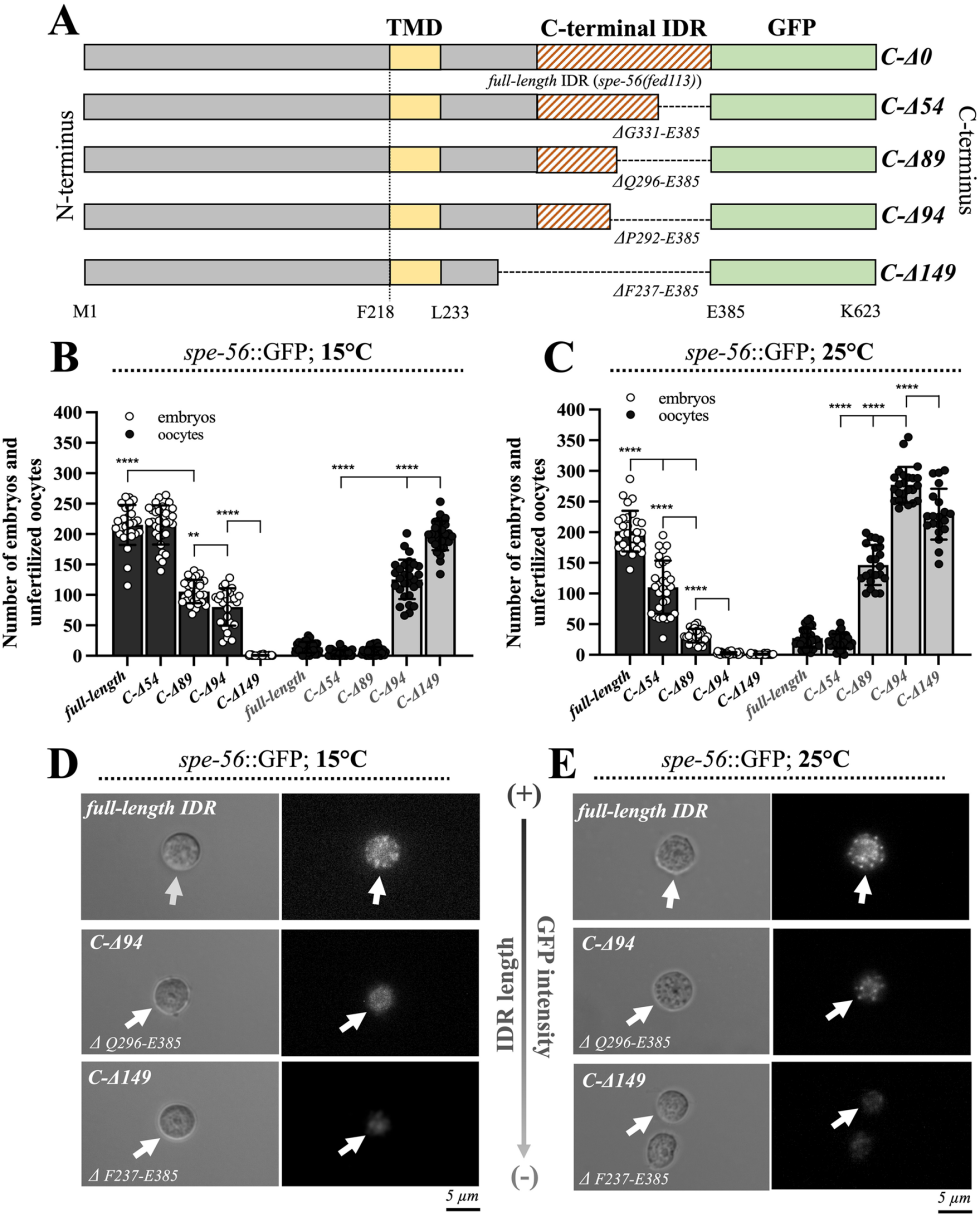
Temperature-dependent fertility of *spe-56* mutant sperm with C-terminal IDR deletions. **(A)** Schematic representation of CRISPR/Cas9-engineered C-terminal deletion alleles (C-Δ) in the *spe-56*::*GFP (allele fed113)* background. Numbers denote amino acid positions flanking the transmembrane domain (TMD), the intrinsically disordered region (IDR), the C-terminal GFP tag, and the respective deletion sites. **(B**,** C)** Quantification of embryos (black bars) and unfertilized oocytes (gray bars) laid by *spe-56*::*GFP* hermaphrodites harboring full-length or truncated (C-Δ) IDRs at 15 °C ***(B)*** and 25 °C ***(C)***. The values represent the means (± SD) of *N* = 3 independent experiments involving *n* ≥ 10 animals per trial. *p < 0.0332; **p < 0.0021; ***p < 0.0002; ****p < 0.0001 (Ordinary one-way ANOVA). **(D**,** E)** Differential interference contrast (DIC) and fluorescence microscopy images of single *spe-56(fed113)* spermatids carrying SPE-56::GFP with either the full-length C-terminal IDR or CRISPR/Cas9-engineered IDR deletion variants (*C-Δ94* or *C-Δ149*), grown at 15 °C **(D)** or 25 °C **(E)**. White arrows indicate spermatids. The gray long arrow between the images indicates the relative loss of GFP fluorescence with increasing IDR deletion.

The incorrect Figures and their accompanying legends appear below.

The original version of this Article has been corrected.

