# Supplementary material for: The intrinsically disordered protein SPE-56 is required for acrosomal-like exocytosis and fertility in Caenorhabditis elegans
Source: Sci Rep. 2026 Apr 11;16:12062. doi: 10.1038/s41598-026-47896-7 (PMC13070043; doi:10.1038/s41598-026-47896-7)
Supplement: Supplementary file 1 — Supplementary Material 1 [file 41598_2026_47896_MOESM1_ESM.docx]

**Supporting Information for**

**The intrinsically disordered protein SPE-56 is required for acrosomal-like exocytosis and fertility in *Caenorhabditis elegans***

**Dieter-Christian Gottschling**^1^* **, Sarah Eiser**^1^**, and Frank Döring**^1^

^1^University of Kiel, Department of Molecular Prevention, Kiel, 24118, Germany

*gottschling@molprev.uni-kiel.de

**Fig. S1**

**Figure S1. Assessment of *in vitro* activation in spe-56-deficient sperm.**
DIC and fluorescence images of *in vitro*-activated spermatozoa isolated from N2 wild-type and *spe-56(t1791)* mutant males. Various chemical agents were used to induce activation, including Triethanolamine **(**TEA; ***a–d*)**, Monensin **(*e–h*)**, Proteinase-K **(*i–l*)**, Pronase **(*m–p*)**, and Zinc **(*r–u*)**, which target different activation mechanisms, including intracellular *p*H-raise, an SPE-8–independent pathway, and the canonical SPE-8 pathway. Black arrows indicate the pseudopods of the activated spermatozoa; white arrows indicate the MO fusion pores. Gray arrows indicate the short pseudopods that are typical of *spe-56-*deficient spermatozoa.

**Fig. S2**

**Figure S2. Multiple sequence alignment of SPE-56 homologs of rhabditid nematodes.**

Amino acid alignments with *Clustal Omega* and *Clustal* ^113^ color scheme shows sequence similarities in *C. elegans* compared with *C. brigsae*, *C. japonica*, *C. remanei*, and *C. brenneri*. The black border indicates disorder prediction by MobiDB ^111^*.*

**Fig. S3**

**Figure S3. Temperature- and IDR-dependent segregation of SPE-56 in C-terminal deletion mutants.**
DIC and fluorescence images of spe-56(fed113) spermatid groups carrying SPE-56::GFP with either the full-length C-terminal IDR or CRISPR/Cas9-engineered IDR-deletion variants (*C-Δ94* or *C-Δ149* ), grown at 15 °C **(A)** or 25 °C **(B)**. Autofluorescence-control images depicting N2 wild-type spermatids are shown below. White arrows indicate spermatids. The gray long arrow between the images indicates the relative loss of GFP fluorescence with increasing IDR deletion.

**Fig. S4**

**Figure S4. Localization patterns of SPE-56::GFP, MOs, and MSP in groups of spermatids and spermatozoa.**
**A.** Immunofluorescence of *spe-56(fed113)* groups of spermatids and spermatozoa carrying SPE-56::GFP performed according to the regular method (Triton X permeabilization; see Methods section). This method depicts antibody staining of MOs (red), SPE-56::GFP (green), and MO/SPE-56::GFP colocalization (yellow). In contrast, immunofluorescence without TritonX 100 permeabilization **(B)** revealed no fluorescence in spermatids but exclusively anti-MO fluorescence (red) in spermatozoa. This indicates that the GFP-labeled SPE-56 C-terminus is intracellular. **(C)** Immunostaining using anti-MSP antibodies (red) was used to visualize cell polarization and localize the Major Sperm Protein (MSP) in pronase-activated spermatozoa isolated from *spe-56(fed116)* null mutants and *spe-56(fed113)* control animals.

**Fig. S5**

**Figure S5. Western blot analysis of SPE-56::GFP.**

**Anti-GFP immunoblot analysis of sperm and whole-worm lysates (lanes 3, 4, 8 and 10) in the presence (lanes 2–6) or absence (lanes 7–10) of GFP-Trap. Lane 1, molecular weight markers (kDa). Lane 2, lysate from *fem-3(q96)* MOG-hermaphrodites. Lanes 3 and 4, whole-worm lysates from *spe-56(t1791)/n*T1 hermaphrodites expressing pharyngeal GFP under the *myo-2* promoter (27 kDa control band). Lanes 5 and 6, and lane 9, sperm lysates from *fem-3(q96),spe-56(fed113)* hermaphrodites (two independent samples) expressing SPE-56::GFP (predicted molecular weight 71 kDa; indicated by black arrows). The predicted molecular weight of native SPE-56 (44 kDa; barely detectable in the absence of GFP fusion) is indicated at right. See Methods section for details.**

**Fig. S6**

**Figure S6. Quantitative effects of temperature on spermatid number and mating efficiency.**

**A. Number of spermatids isolated from N2 wild-type males and *spe-56(t1791)* mutant males raised at 15 °C or 25 °C. B. Young adult *spe-56(C-Δ94)* mutant males and early L4 *fog-2(q71)* spermless hermaphrodites were isolated from mixed populations grown at 15 °C or 25°C under *ad libitum* feeding and placed on separate 35-mm agar plates for 24 h, subsequently mated overnight at either 15 °C or 25 °C (10 males : 2 hermaphrodites) and then separated on individual plates. Progeny and unfertilized oocytes were counted daily for a period of 3 days on each plate.**The values represent the means (± SD) of N = 3 independent experiments involving n ≥10 animals per trial. *p < 0.0332; **p < 0.0021; ***p < 0.0002; ****p < 0.0001 (Ordinary one-way ANOVA).
